# *Codonopsis pilosula*-derived glycopeptide dCP1 promotes the polarization of tumor-associated macrophage from M2-like to M1 phenotype

**DOI:** 10.1007/s00262-024-03694-6

**Published:** 2024-05-14

**Authors:** Hongxu Liu, Maojin Yao, Jiaoyan Ren

**Affiliations:** 1 School of Food Science and Engineering, South China University of Technology, Guangzhou, 510641 Guangdong People’s Republic of China; 2 State Key Laboratory of Respiratory Diseases, The First Affiliated Hospital of Guangzhou Medical University, Guangzhou, 510120 Guangdong People’s Republic of China

**Keywords:** Tumor-associated macrophage, Glycopeptide, Phenotype polarization, Lipid metabolism, Structure–activity relationship

## Abstract

The majority of the immune cell population in the tumor microenvironment (TME) consists of tumor-associated macrophages (TAM), which are the main players in coordinating tumor-associated inflammation. TAM has a high plasticity and is divided into two main phenotypes, pro-inflammatory M1 type and anti-inflammatory M2 type, with tumor-suppressive and tumor-promoting functions, respectively. Considering the beneficial effects of M1 macrophages for anti-tumor and the high plasticity of macrophages, the conversion of M2 TAM to M1 TAM is feasible and positive for tumor treatment. This study sought to evaluate whether the glycopeptide derived from simulated digested *Codonopsis pilosula* extracts could regulate the polarization of M2-like TAM toward the M1 phenotype and the potential regulatory mechanisms. The results showed that after glycopeptide dCP1 treatment, the mRNA relative expression levels of some M2 phenotype marker genes in M2-like TAM in simulated TME were reduced, and the relative expression levels of M1 phenotype marker genes and inflammatory factor genes were increased. Analysis of RNA-Seq of M2-like TAM after glycopeptide dCP1 intervention showed that the gene sets such as glycolysis, which is associated with macrophage polarization in the M1 phenotype, were significantly up-regulated, whereas those of gene sets such as IL-6-JAK-STAT3 pathway, which is associated with polarization in the M2 phenotype, were significantly down-regulated. Moreover, PCA analysis and Pearson's correlation also indicated that M2-like TAM polarized toward the M1 phenotype at the transcriptional level after treatment with the glycopeptide dCP1. Lipid metabolomics was used to further explore the efficacy of the glycopeptide dCP1 in regulating the polarization of M2-like TAM to the M1 phenotype. It was found that the lipid metabolite profiles in dCP1-treated M2-like TAM showed M1 phenotype macrophage lipid metabolism profiles compared with blank M2-like TAM. Analysis of the key differential lipid metabolites revealed that the interconversion between phosphatidylcholine (PC) and diacylglycerol (DG) metabolites may be the central reaction of the glycopeptide dCP1 in regulating the conversion of M2-like TAM to the M1 phenotype. The above results suggest that the glycopeptide dCP1 has the efficacy to regulate the polarization of M2-like TAM to M1 phenotype in simulated TME.

**Supplementary Information:**

The online version contains supplementary material available at 10.1007/s00262-024-03694-6.

## Introduction

The doctrine that the immune system is the main defense mechanism against tumor due to its ability to recognize and destroy tumors has been generated for decades [1]. Although the immune system can prevent tumor development, it can also support tumor growth and metastasis by being modified by the tumor. The interaction between immune cells and tumor cells is known as cancer immunoediting. During cancer immunoediting, the host immune system shapes the tumor fate in three stages (elimination, homeostasis, and escape) by activating intrinsic and acquired immune mechanisms. In the elimination phase, transformed cells are destroyed by a competent immune system. The sporadic tumor cells that escape immune destruction may then enter the equilibrium phase, where editing occurs. The escape phase represents the final stage of the process, in which the immunologically ‘sculpted’ tumor starts to grow gradually and establishes an immunosuppressive tumor microenvironment that is conducive to its own growth [2]. Therefore, research and development that can effectively modulate the immune system and thus enhance its ability to recognize and attack tumor cells is a promising research direction for cancer therapy.

Studies have shown that natural products, including polysaccharides, polypeptides, alkaloids, saponins, and flavonoids, have immunomodulatory effects [3]. Among them, polysaccharides are one of the most widely studied components. Polysaccharides have been shown to perform immunomodulation by promoting immune organ development, regulating immune cell activity, and secretion of immune-related molecules, such as *β*-glucan, which is used in clinical. The regulatory effects of polysaccharides on immunity can be divided into direct and indirect effects [4]. The direct effect is that the polysaccharide directly acts on the immune system and immune cells, while the indirect action is the metabolism of polysaccharides by intestinal microorganisms to short-chain fatty acids and their utilization by immune cells. Considering their large molecular weight, most polysaccharides exert their immunomodulatory effects indirectly. Another potential immunomodulator is polypeptide, and one of the examples of successful application in immune regulation is thymosin. The immunomodulatory effects of peptides can enter cells by directly affecting receptors (e.g., TLRs) or through passive diffusion, peptide transporters, and fluid phase endocytosis and subsequently interfere with inflammatory signaling pathways such as NF-*κ*B, MAPK, and PI3K signaling pathway [5].

Glycopeptides are peptides that contain carbohydrate moieties (glycans) covalently attached to the side chains of the amino acid residues that constitute the peptide. Compared to polysaccharides, glycopeptides have a small molecular weight; compared to polypeptides, the glycan chains contained in them are recognized by cellular receptors as antigenic epitopes and the structural changes caused by glycosylation modification make them obtain higher immunogenicity. These characteristics make it a potential candidate for immunomodulatory. Studies show natto glycopeptides extracted from natto can better elevate IFN-γ expression in splenic lymphocytes [6]; the glycoproteins (DOT) in *Dioscorea opposita* Thunb exert its immunomodulatory activity by increasing the production of TNF-*α*, IL-6, and NO in macrophages and enhancing pinocytosis through mitogen-activated protein kinase and NF-*κ*B signaling pathway [7]. Glycoprotein from *Oldenlandia diffusa* enhances IL-1 and TNF-*α* production by monocytes and enhances their phagocytosis of tumor cells [8]. These studies suggest that glycopeptides have immunomodulatory functions.

In our previous study, it was found that the *Codonopsis pilosula* extracts have immunomodulatory effects, and the polysaccharide obtained from it has the effect of regulating TAM phenotype on melanoma-bearing mouse model. Also as the components in *Codonopsis pilosula* extracts, we speculated whether glycopeptide also has the effect of regulating TAM phenotype. Therefore, we studied the effects of *Codonopsis pilosula* glycopeptide on TAM phenotypic polarization in low levels of glucose, acidic, and lipid-rich simulated tumor microenvironments and explored the possible mechanisms by which glycopeptide dCP1 affects TAM phenotype polarization through RNA-Seq and lipid metabolism, and attempt to explain the relationship between its structure and function through molecular dynamics simulations.

## Methods

### Reagents and materials

Mouse melanoma cell line B16 was purchased from the Chinese Academy of Sciences (Shanghai, China). RAW264.7 cells were gracefully provided by Prof. Zhaoyu Liu of Sun Yat-Sen Memorial Hospital (Sun Yat-sen University, Guangzhou, China). *Codonopsis pilosula* polysaccharide (dCPP) and glycopeptide (dCP1) were isolated and purified by our laboratory. PrimeScript™ RT Reagent Kit with gDNA Eraser (RR047A) and TB Green™ Premix Ex Taq™ II (Tli RNaseH Plus) (RR820A) were purchased from TAKARA. Methanol (A454-4), acetonitrile (A996-4), Roswell Park Memorial Institute (RPMI) 1640 Medium (C11875500BT), Dulbecco's Modified Eagle's Medium (DMEM) (31600034), and Qubit® ssDNA Assay Kit were purchased from Thermo Fisher Scientific, USA. BGI Optimal mRNA Library Construction Kit (LR00R96), BGI Plug-In Adapter Kit (LA00R04), and DNA sorting beads (LB00V60) were purchased from BGI Genomics. MGISEQ-2000RS high-throughput sequencing reagent kit (FCL PE150) (1000012555) was purchased from MGI Tech Co., Ltd, Shenzhen. Lipopolysaccharide LPS (L6529) and bovine serum albumin (B2064) were purchased from Sigma-Aldrich, USA. Fetal bovine serum (04-001-1A-US) was purchased from BI, ISR. Stearic acid (S815203) and oleic acid (O815203) were purchased from Macklin Biochemical Technology Co., Shanghai. Soft fatty acid (P101061) and linoleic acid (L100442) were purchased from Shanghai Aladdin Reagent Co. Sephadex G-100 (S14034) was purchased from Yuanye Bio., Shanghai. Recombinant mouse interleukin 4 (214-14) was purchased from PeproTech, USA. Protein sequencing grade trypsin (V5111) was purchased from Promega, USA. WGP (tlrl-wgp) was purchased from InvivoGen, France. Standard sensitivity RNA analysis kit (DNF-47-0500) was purchased from Agilent, USA. Formic acid ammonia (17843-250G) was purchased from Honeywell Fluka, USA.

### Isolation and purification of *Codonopsis pilosula-*derived glycopeptides dCP1

*Codonopsis pilosula* extracts were obtained according to the previous method [9], and simulated digestion was performed to obtain the digested product dCPCP, which was further purified by Sephadex G-100 to obtain high purity glycopeptides. Elution was performed with deionized water at a flow rate of 300 μL/min, and 3.6 mL of eluate was collected from each tube. Phenol–sulfuric acid and BCA were used to determine the total carbohydrate and protein content in each tube. The overlapping symmetric peaks were collected and then concentrated and lyophilized to obtain the purified glycopeptide dCP1.

### UV spectral identification of dCP1

5.0 mg of dCP1 was dissolved in 10 mL of 0.4 mol/L NaOH solution in a water bath at 60 °C for 30 min. after which it was scanned using a UV spectrophotometer at wavelengths between 190 and 350 nm. Under the same conditions, dCP1 untreated with 0.4 mol/L NaOH solution was used as a control and scanned. Whether dCP1 has an O-glycopeptides bond was determined based on whether there is an absorbance change at 240 nm [10].

### LC–MS/MS detection of dCP1

A capillary high-performance liquid chromatograph (Ultimate 3000) coupled with a tandem electrospray-combined ion trap Orbitrap mass spectrometer was used for the separation and detection of glycopeptides. The specific conditions are described as follows:

- *Chromatographic conditions* The chromatographic column consisted of a pre-column and an analytical column. The pre-column was a C18 column (300 μm i.d. × 5 mm, packed with Acclaim PepMap RPLC C18, 3 μm, 100 Å), and the analytical column was a C18 column (150 μm i.d. × 150 mm, packed with Acclaim PepMap RPLC C18, 1.9 μm, 100 Å); mobile phase A was 0.1% formic acid solution (Liquid A), and mobile phase B was 80% ACN + 0.1% formic acid solution.
- *Gradient elution conditions* Each component was analyzed for 120 min. 0–5 min, 4–10% mobile phase B; 5.1–85 min, 10–22% mobile phase B; 85.1–110 min, 22–40% mobile phase B; 110.1–115 min, 40–95% mobile phase B; 115.1–120 min, 95% mobile phase B. The flow rate was 600 nL/min, and the injection volume was 5 μL.
- *Mass spectrometry conditions* EThcD mode was used for fragmentation, and the primary mass spectrometry conditions were Orbitrap resolution = 60,000, mass-to-nucleus ratio range of 375–2000 m/z for mass spectrometry scan, maximum injection time of 54 ms, and AGC of 800,000; secondary mass spectrometry conditions were Resolution = 30,000, maximum injection time of 54 ms, AGC of 100,000, NCE of 32, and Orbitrap detector.

The MS raw files were searched in Swiss-Prot (reviewed) database using Byonic (v4.2.4) software.

### Preparation of degreasing serum

To prevent fatty acids in the serum from interfering with the experiments, the serum was defatted. The method was modified slightly according to the literature [11, 12] by adding 360 mg of silica powder per milliliter of fetal bovine serum, oscillating at low temperature for 2 h, followed by centrifugation at 10,000 rpm/min at 4 °C for 10 min and then collecting the supernatant, adding 360 mg of silica per mL of serum to the supernatant, and incubating at 4 °C for 12 h, then centrifuged under the same conditions to obtain the defatted serum.

### Preparation of BSA blank carrier

0.02 mmol fatty acid-free BSA was dissolved in 3800 μL DMEM basal medium, followed by 200 μL anhydrous ethanol and stored at −20 °C. When used, dilute so that the final concentration of BSA is 0.4 mmol/L and the volume fraction of ethanol is 0.4%.

### Preparation of BSA-FFA complex

FFA cannot be absorbed by cells without binding carrier proteins. Numerous studies have found that the most abundant FFAs in the TME include stearic, palmitic, oleic, and linoleic acids [13–20]. Therefore, the above four FFAs were selected to combine with BSA to form a complex to simulate the lipid-rich TME environment. The preparation method of the BSA-FFA complex was referred to relevant literature and slightly modified [21–31]. The specific method was as follows: DMEM basic medium was used to dissolve non-fatty acid BSA, and anhydrous ethanol was used to dissolve fatty acid so that the molar ratio of BSA: FFA = 1:5. 0.02 mmol fatty acid-free BSA was dissolved in 3800 μL DMEM base medium, and 0.1 mmol each of oleic acid, linoleic acid, stearic acid, and palmitic acid were dissolved in 200 μL anhydrous ethanol, respectively. Stearic acid and palmitic acid were dissolved in the ultrasonic water bath at 50 °C. BSA solution was mixed with FFA solution and incubated for 4 h at 37 °C under vigorous shaking to promote the coupling of fatty acids and BSA. The reserve solution is stored at −20 °C. The above solutions should be filtered by a 0.22 μm sterile filter. When diluted, the final FFA concentration in the four kinds of BSA-FFA complexes was 0.5 mmol/L and the final BSA concentration was 0.1 mmol/L.

### Low-glucose, low-serum, lipid-rich, acidic complete medium preparation

DMEM medium (low glucose) was used as the basal medium, and 20 mmol sodium bicarbonate was added per liter of basal medium. The complete medium was prepared by adding BSA-FFA complex and 3% defatted serum. Adjust the pH value of the complete medium to 6.7 by 1 mol/L HCl and 1 moL/L NaOH.

### Cell culture and grouping

B16 cells were cultured with complete medium until the confluency reached 90%, then replaced with serum-free basal medium, and continued to culture for 24 h, after which the supernatant was collected as conditioned medium (CM) that simulated the tumor microenvironment. RAW264.7 cells were cultured in 6-well plates, and the number of cells inoculated in each well was 1 × 10^4^. After the cells were cultured in complete medium containing 5% FBS high glucose DMEM for a period of time, the cells were cultured in the following groups, and three repeated experiments were set in each group. The specific experimental grouping and processing methods are as follows:

- *Group A1* RAW264.7 were cultured in complete medium with high glucose DMEM containing 10% normal serum for 8 days, after which cells were collected for further experiments.
- *Group A2* RAW264.7 were cultured with high-glucose DMEM complete culture medium containing 10% normal serum for 8 days and cultured with LPS (100 ng/mL) complete culture medium for 24 h after 7 days to polarize them into M1 phenotype, after which cells were collected for further experiments.
- *Group A3* RAW264.7 were cultured with high-glucose DMEM complete medium containing 10% normal serum for 8 days. After 7 days, cells were stimulated with IL-4 (100 ng/mL) complete medium for 24 h to polarize into M2 phenotype, after which cells were collected for further experiments.
- *Group B1-1* RAW264.7 were cultured using low glucose, low serum, lipid-rich, and acidic complete medium mixed with CM at 1:1, and the final concentration of defatted serum was 1.5% and the final concentration of FFA was 0.5 mmol/L. The medium was changed every 2–3 days, and the cells were cultured for a total of 8 days, after which cells were collected for further experiments.
- *Group B1-2* RAW264.7 were cultured using low glucose, low serum, lipid-rich and acidic complete medium mixed with CM at 1:1, and the final concentration of defatted serum was 1.5% and the final concentration of FFA was 0.5 mmol/L. The cells were cultured for a total of 8 days, and the medium was changed every 2–3 days. After 7 days of culture, the cells were stimulated with complete medium containing LPS (100 ng/mL) for 24 h to polarize into M1-like TAM phenotype, after which the cells were collected for further experiments.
- *Group B1-3* RAW264.7 were cultured using low glucose, low serum, lipid-rich, and acidic complete medium mixed with CM at 1:1, and the final concentration of defatted serum was 1.5% and the final concentration of FFA was 0.5 mmol/L. The cells were cultured for 8 days, and the medium was changed every 2–3 days. After 7 days of culture, the cells were stimulated with complete medium containing IL-4 (100 ng/mL) for 24 h to polarize into M2-like TAM phenotype, after which the cells were collected for further experiments.
- *Group C1-1* RAW264.7 were cultured using low glucose, low serum, lipid-rich and acidic complete medium mixed with CM at 1:1, so that the final concentration of defatted serum was 1.5% and the final concentration of FFA was 0.5 mmol/L. The cells were cultured for 8 days, and the medium was changed every 2–3 days. After 7 days of culture, complete medium containing dCP1 (200 μg/mL) was used to stimulate for 24 h, and cells were collected for further experiments.
- *Group C1-2* RAW264.7 were cultured using low glucose, low serum, lipid-rich, and acidic complete medium mixed with CM at 1:1, and the final concentration of defatted serum was 1.5% and the final concentration of FFA was 0.5 mmol/L. The cells were cultured for 8 days and the medium was changed every 2–3 days. After 7 days, the cells were stimulated with complete medium containing LPS (100 ng/mL) for 24 h to polarize into M1-like TAM phenotype and then stimulated with dCP1 (200 μg/mL) for 24 h, after which cells were collected for further experiments.
- *Group C1-3* RAW264.7 were cultured using low glucose, low serum, lipid-rich and acidic complete medium mixed with CM at 1:1, and the final concentration of defatted serum was 1.5% and the final concentration of FFA was 0.5 mmol/L. The cells were cultured for 8 days, and the medium was changed every 2–3 days. After 7 days, the cells were stimulated with complete medium containing IL-4 (100 ng/mL) for 24 h to polarize into M2-like TAM phenotype and then treated with dCP1 (200 μg/mL) for 24 h, after which the cells were collected for further experiments.
- *Group D2* RAW264.7 were mixed with low glucose, low serum, lipid-rich, and acidic complete medium at 1:1 with CM, and the final concentration of defatted serum was 1.5% and the final concentration of FFA was 0.5 mmol/L. The cells were cultured for 7 days, and the medium was changed every 2–3 days. After 7 days of culture, the cells were stimulated with complete medium containing IL-4 (100 ng/mL) for 24 h to polarize into M2-like TAM phenotype and then treated with dCPP (200 μg/mL) for 24 h, after which the cells were collected for further experiments.
- *Group E (WGP positive drug control)* RAW264.7 were cultured using low glucose, low serum, lipid-rich, acidic complete medium mixed with CM at 1:1, and the final concentration of defatted serum was 1.5%, and the final concentration of FFA was 0.5 mmol/L. The cells were cultured for 7 days, and the medium was changed every 2–3 days. After 7 days of culture, the cells were stimulated with complete medium containing IL-4 (100 ng/mL) for 24 h to polarize into M2-like TAM phenotype and then treated with WGP (100 ng/mL) for 24 h, after which cells were collected for further experiments.
- *Group F (carrier control)* RAW264.7 were cultured with low glucose, low serum, lipid-rich, and acidic complete medium mixed with CM at 1:1, and the final concentration of defatted serum was 1.5%, and the final concentration of carrier BSA was 0.4 mmol/L. The cells were cultured for 8 days, and the medium was changed every 2–3 days. After 7 days, the cells were stimulated with IL-4 (100 ng/mL) or LPS (100 ng/mL) for 24 h, after which the cells were collected for further experiments.

### Quantitative real-time fluorescence PCR

Total RNA was extracted from cultured cells using TRIzol reagent and detected by qRT-PCR using the CFX96 real-time PCR detection system. The primers used in this study are shown in Supplementary Table S1. β-actin was used as an internal reference, and the relative mRNA expression levels were calculated by the 2^−ΔΔCt^ method.

### RNA-Seq and analysis

RNA-Seq analysis was performed on groups A1, A2, A3, B1-1, B1-2, B1-3, C1-1, C1-2, C1-3, and D2. RNA was extracted using TRIzol followed by mRNA Library Preparation (DNBSEQ) and sequenced. The raw data obtained from sequencing were filtered using SOAPnuke (v1.5.6) [32] to obtain clean data. Clean data were matched to the reference genome using HISAT2 (v2.1.0) [33] software. The clean data were compared to the reference gene set using Bowtie2 (v2.3.4.3). Gene expression quantification was performed using RSEM (v1.3.1) [34] software and heat map of gene expression clustering in different samples using pheatmap (v1.0.8) [35]. Differential gene detection was performed using DESeq2 (v1.4.5) [36] with *q* value ≤ 0.05 or FDR ≤ 0.001. Further in-depth exploration of gene functions associated with phenotypic changes was performed. Based on hypergeometric tests, KEGG enrichment analysis of differential genes was performed using Phyper with *q* value ≤ 0.05 as the threshold [37], and those meeting this condition were defined as significantly enriched in candidate genes.

### Metabolite extraction and UPLS-MS/MS analysis

Groups B1-2, B1-3, C1-2, and C1-3 were selected for subsequent lipid metabolomics study. The cells were removed from the incubator; the medium was decanted and washed 3 times with 1 × PBS. Afterward, cells were scraped with a cell scraper, carefully transferred to a freezing tube, and frozen in liquid nitrogen until use.

For metabolite extraction, cells were transferred from the lyophilization tube to a 2-mL thickened centrifuge tube, and 2 small magnetic beads were added. 800 μL of pre-chilled dichloromethane/methanol (3:1, V/V) precipitant was added, and 10 μL of internal standard was added to each sample. Afterward, the samples were ground using a tissue grinder for 5 min and sonicated in an ice bath for 10 min. After resting overnight in a refrigerator at −20 °C, the samples were centrifuged at 25,000 g/min for 15 min using a centrifuge at 4 °C. 600 μL of supernatant was taken and concentrated and lyophilized in a freeze concentrator. After lyophilization, the samples were reconstituted with 120 μL of lipid re-soluble solution (isopropanol: acetonitrile: water = 2:1:1) and shaken for 10 min. After that, the samples were sonicated in an ice bath for 10 min and centrifuged at 25,000 g/min for 15 min at 4℃ to obtain the samples. 20 μL of each sample was taken and prepared for UPLC-MS/MS analysis. A UPLC I-Class Plus (Waters, USA) tandem with a Q Exactive high-resolution mass spectrometer (Thermo Fisher Scientific, USA) was used for the separation and detection of metabolites. The specific experimental conditions are described as follows:

A UPLC I-Class Plus (Waters, USA) tandem with a Q Exactive high-resolution mass spectrometer (Thermo Fisher Scientific, USA) was used for the separation and detection of metabolites. The specific experimental conditions were as follows:

- *Chromatographic conditions* The chromatographic column was a CSH C18 column (1.7 μm 2.1*100 mm, Waters, USA). The mobile phase A was 60% acetonitrile + 10 mM ammonium formate + 0.1% formic acid (liquid A) in positive ionization mode, and the mobile phase B was 90% isopropanol + 10% acetonitrile + 10 mM ammonium formate + 0.1% formic acid; the mobile phase A was 60% acetonitrile + 10 mM ammonium formate in negative ionization mode, and the mobile phase B was 90% isopropanol + 10% acetonitrile + 10 mM ammonium formate.
- *Gradient elution conditions* 0–2 min, 40–43% mobile phase B; 2–2.1 min, 43%–50% mobile phase B; 2.1–7 min, 50–54% mobile phase B; 7–7.1 min, 54–70% mobile phase B; 7.1–13 min, 70–99% mobile phase B; 13–13.1 min, 99–40% mobile phase B mobile phase B; 13.1–15 min, 40% mobile phase B. The flow rate was 0.4 mL/min, the column temperature was 55 °C, and the injection volume was 5 μL.
- *Mass spectrometry conditions* mass spectrometry scan mass-to-nucleus ratio range of 200–2000, primary resolution of 70,000, AGC of 300,000, and maximum injection time of 100 ms. According to the parent ion intensity, Top3 was selected for fragmentation, and secondary information was collected with secondary resolution of 17,500, AGC of 100,000, and maximum injection time of 50 ms. The fragmentation energy (stepped NCE) was set to: 15, 30, 45 eV.
- *The ion source (ESI) parameters were set* sheath gas flow rate of 40, auxiliary gas flow rate of 10, spray voltage (|KV|) of 3.80 for positive ion mode and 3.20 for negative ion mode, ion transport tube temperature of 320 °C, and auxiliary gas heating temperature of 350 °C. The ion source (ESI) parameters were set: sheath gas flow rate of 40, auxiliary gas flow rate of 10, spray voltage (|KV|) of 3.80 for positive ion mode and 3.20 for negative ion mode.

The obtained mass spectrometry data were imported into LipidSearch v.4.1 (Thermo Fisher Scientific, USA) software for mass spectrometry data analysis, and then, a data matrix containing information such as lipid molecule identification results and quantitative results was obtained and processed for information analysis. The specific parameters are as follows:

The search library mass deviation of precursor ions and product ions was 5 ppm; the response threshold was the relative response deviation of product ions of 5.0%; the peak lift mass deviation was 5 ppm; the M-score was 5.0; the c-score was 2.0; the positive ion mode additive forms were [M + H]^+^, [M + NH4]^+^, [M + Na]^+^, and the negative ion mode additive forms were [M–H]^−^, [M–2H]^−^, [M + HCOO]^−^; retention time deviation of 0.1 min.

### Alkylation, enzymatic hydrolysis, and desalination of dCP1

After denaturing the dCP1 sample with urea/ammonium bicarbonate buffer, 15 mg of dCP1 was added to a certain amount of 1 mol/L dithiothreitol (DTT) solution to a final DTT concentration of 10 mmol/L and reduced in a water bath at 56 °C for 1 h. Afterward, 10 μL of 0.5 mol/L iodoacetamide (IAA) solution was added to a final IAA concentration of 55 mmol/L, and the reaction was carried out at room temperature and protected from light for 40 min. After that, 2 μL of 1 mol/L DTT solution was added to make the final concentration of DTT 20 mmol/L to neutralize the unreacted IAA.

The alkylated samples were adjusted to pH 8.0 by ammonium bicarbonate solution, and trypsin was added according to the mass ratio of trypsin to a substrate of 1:100. The samples were digested using trypsin at 37 °C for 4 h, and then, the pancreatin was added according to the mass ratio of 1:100, and the enzymolysis reaction was conducted at 37℃ overnight (16 h). The sample solution was adjusted to pH < 2 by trifluoroacetic acid (TFA) and then centrifuged at 13,000 rpm for 15 min; supernatant was taken to prepare for desalting with C18 solid phase extraction column. The C18 extraction column was activated with ≥ 99.9% acetonitrile, followed by liquid replacement with 50% acetonitrile /0.1% TFA, balanced with 2% acetonitrile /0.1% TFA solution; then, samples were taken and then cleaned with 2% acetonitrile /0.1% TFA solution. Finally, 50% acetonitrile /0.1%TFA eluent was used to elute the desalted glycopeptides on the chromatographic column, and the solvent was dried in a vacuum centrifugal concentrator at 45 °C. The glycopeptides were fully dissolved with the sample solution (0.1% formic acid, 2% acetonitrile), then whirled, and centrifuged at 13,200 rpm for 10 min at 4 °C. The supernatant was transferred to sample tubes for LC–MS/MS detection and analysis.

### Molecular docking and molecular dynamics simulations

Amino acid sequences of each glycopeptide were obtained from glycopeptides LC–MS/MS data, and the spatial structure of the peptide was predicted using ColabFold v1.5.2 [38] algorithm based on AlphaFold2, and the three-dimensional structure data pdb file with the highest prediction score of the corresponding peptide segment was obtained. Carbohydrate chain composition and glycosylation information were obtained according to the glycopeptides LC–MS/MS data. Suitable carbohydrate chain was screened from Glyconnect [39]. Carbohydrate Builder was used to model the carbohydrate chain, and the lowest energy conformation was selected as the predicted carbohydrate chain structure. On this basis, Glycoprotein Builder was used to model glycopeptides and its structure, and the three-dimensional structure data of glycopeptides pdb file were obtained, which could be used for the further study of receptor–ligand interaction. The three-dimensional structure data pdb files of receptor protein crystal that can recognize and bind glycopeptides or glycoproteins expressed in murine macrophages were searched from RCSB [40] and AlphaFold Protein Structure Database [41].

Chimera 1.1.6 was first used to hydrogenate the receptors and glycopeptides [42], followed by a preliminary screening of glycopeptides binding to receptors using ZDOCK Server 3.0.2 [43]. GROMACS 2021.5 software package was used to simulate the receptors protein and glycopeptides molecular dynamics. The Amber14SB force field was used [44]. TIP3P-dominant water model was chosen, and periodic boundary conditions were set. The workflow of molecular dynamics simulation includes four steps: energy minimization, NVT equilibrium, NPT equilibrium, and production dynamics simulation. Firstly, the protein and ligand heavy atoms were constrained to minimize the energy of water molecules by 5000 steps via the steepest descent method. Then, maintaining the constraints, a 50,000-step NVT ensemble simulation was carried out for the whole system. The temperature was 298 K, and the time step was 2 fs. Finally, the molecular dynamics simulation of the system was carried out in the NPT ensemble for 100 ns with a time step of 2 fs. The relevant parameters were analyzed by the module of the GROMACS software package. The binding energy was analyzed via g_mmpbsa.sh [45].

### Statistical analysis

All statistical analyses were processed using SPSS 22 (IBM Corporation, Armonk, New York, USA). Data are expressed as mean ± standard deviation. In the statistical analysis, the Student’s t test was used to analyze the significance between two groups, and the one-way analysis of variance was used to evaluate the differences among multiple groups. The Duncan test was used when the variances were homogeneous, and the Welch’s Anova test was used when the variances were not homogeneous. *p* < 0.05 was considered significant. The results were plotted using GraphPad Prism 8 and Origin 2022 software.

## Results

### dCP1 is a mixed glycopeptide containing a large number of O-glycosylated peptides

**Fig. 1:**
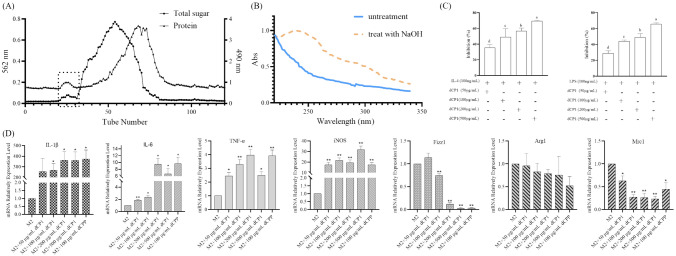
Purification of glycopeptide dCP1 and its effect on TAM activity and phenotypic changes. (**A**) Purification of dCP1 by Sephadex G-100 chromatography; (**B**) UV spectrum of dCP1; (**C**) Effects of dCP1 on the proliferation of M1/M2-like TAM; different lowercase letters indicate significant differences (*p* < 0.05); (**D**) Effects of different concentrations of dCP1 on relative mRNA expression levels of M1/M2 marker genes in M2-like TAMs. *indicates *p* < 0.05, **indicates *p* < 0.01, ***indicates *p* < 0.001

The digested dCPCP was purified by Sephadex G-100 column chromatography. It was found that only 21–31 tubes in the deionized water eluent had the carbohydrates peak coinciding with the protein peak and symmetrical (dCP1), indicating that dCP1 is a glycoprotein or glycopeptides (Fig. 1A). The carbohydrate content of dCPP was 82.3%, and the protein content was 9.5%. Figure 1B shows that compared with the solid line, the dotted line part, that is, the absorbance of the dCP1 solution treated with NaOH, has a significant change in the absorption peak at 240 nm, indicating that after NaOH treatment, the β-elimination reactions occur. The threonine may be converted into α-aminobutenoic acid, and the serine may be converted into α-aminoacrylic acid. The unsaturated amino acids formed by the two have obvious absorption at 240 nm, so it is preliminary judged that dCP1 contains O-glycopeptides bonds [10, 46, 47]. Then, the complete glycopeptide was characterized; the glycopeptides fractions were firstly separated by capillary high-performance liquid chromatography after enzymatic digestion and desalting, and then fragmented and detected by tandem electrospray ion trap-Orbitrap mass spectrometer, followed by the identification of the amino acid composition of the peptides, the composition of the carbohydrate chains, and the glycosylation sites in the glycopeptides by Byonic system software to complete the structural analysis of the complete glycopeptides. Analysis revealed that dCP1 is a mixture of multiple glycopeptides; 95 kinds of glycosylated peptides were detected, including 9 N-glycosylated glycopeptides and 86 O-glycosylated glycopeptides, indicating that about 9.5% of the glycopeptides have been identified to contain N-glycosylation sites and 90.5% of the glycopeptides have O-glycosylation sites; the length of the peptide chain ranged from 4 to 37 amino acids. Detailed information on glycopeptides is shown in Table S2.

### dCP1 inhibits TAM activity and promotes the polarization of M2-like TAM to M1 phenotype

The preliminary M1/M2-like TAM model was established according to the method in the previous study [9], and then, different concentrations of glycopeptides were used to intervene in order to preliminarily investigate whether glycopeptides affect TAM proliferation and whether they have the function of regulating TAM phenotype. In vitro experiments showed that dCP1 inhibited the proliferation of M1/M2-like TAM in a dose-dependent manner in the concentration range of 50–500 μg/mL, taking M1 or M2-like TAM without dCP1 treatment as a control (Fig. 1C).

To explore whether dCP1 has the potential to repolarize M2-like TAM to M1 phenotype, the relative expression levels of mRNAs of M1/M2 phenotype marker genes and inflammatory factors genes in TAM were measured by qRT-PCR and compared with the effect of 100 μg/mL dCPP treatment that has a regulatory effect on TAM phenotype (Fig. 1D). Mannose receptor type C 1 (MRC1), a membrane-bound protein, is expressed predominantly on the surface of macrophages. Mrc1 is one of the most commonly used markers to recognize M2 macrophages. It has regulatory endocytosis and phagocytosis functions and plays an important role in immune homeostasis by removing unwanted mannoprotein. Compared with M2-like TAM without dCP1 treatment, M2-like TAM cells after dCP1 treatment showed a significant decrease in Mrc1 mRNA relative expression levels with increasing dCP1 concentrations in a dose-dependent manner, with medium to high doses (100, 200, and 500 μg/mL) of dCP1 having a stronger effect on the reduction in Mrc1 mRNA relative expression levels than 100 μg/mL dCPP treatment. Arg1 has an important role in M2 phenotype macrophages, which can inhibit the inflammatory response by regulating the production and release of inflammatory mediators. It can also convert arginine to ornithine and urea. The relative expression level of Arg1 mRNA gradually decreased with the increase in dCP1 level, but none of them was significantly different compared with control. Fizz1 can inhibit inflammatory responses, reduce inflammatory injury, and is one of the markers of the M2 phenotype of macrophages. The relative expression level of Fizz1 mRNA first increased and then decreased, and the relative expression level of Fizz1 mRNA was slightly increased in cells treated with 50 μg/mL dCP1 only, while the relative expression level of Fizz1 mRNA significantly decreased after higher dose of dCP1 treatment. IL-1β is a cytokine secreted by activated M1-type macrophages. It plays an important regulatory role in the inflammatory response. IL-1β promotes the production and release of inflammatory mediators such as pro-inflammatory cytokines (e.g., TNF-α, IL-6, etc.) and participates in the activation of inflammatory signaling pathways. The relative expression levels of IL-1β mRNA were increased after treatment with different doses of dCP1, with the highest increase after treatment with 200 μg/mL dCP1, but lower than the elevated effect of intervention with 100 μg/mL dCPP on the relative expression levels of IL-1β mRNA. iNOS is a key enzyme in the macrophage inflammatory response and is a source of nitric oxide (NO), which is efficiently induced in response to pro-inflammatory stimuli. The relative expression levels of iNOS mRNA were all significantly increased and were all higher than the intervention effect of 100 μg/mL dCPP. IL-6 is a pro-inflammatory factor that can be released by M1 macrophages, the relative expression levels of IL-6 mRNA were all increased and significantly different, but all were lower than the intervention effect of 100 μg/mL dCPP. TNF-α is a cytokine that can be released by M1 phenotype macrophages and has the function of promoting inflammatory response and enhancing immune response. The relative expression level of TNF-α mRNA was also significantly increased, but only the relative expression level of TNF-α mRNA after 200 μg/mL dCP1 treatment was higher than that after 100 μg/mL dCPP intervention.

### *Codonopsis pilosula-*derived polysaccharide dCPP and glycopeptide dCP1 both can affect the relative expression level of M1/M2 marker genes in the simulated tumor microenvironment TAM model

**Fig. 2:**
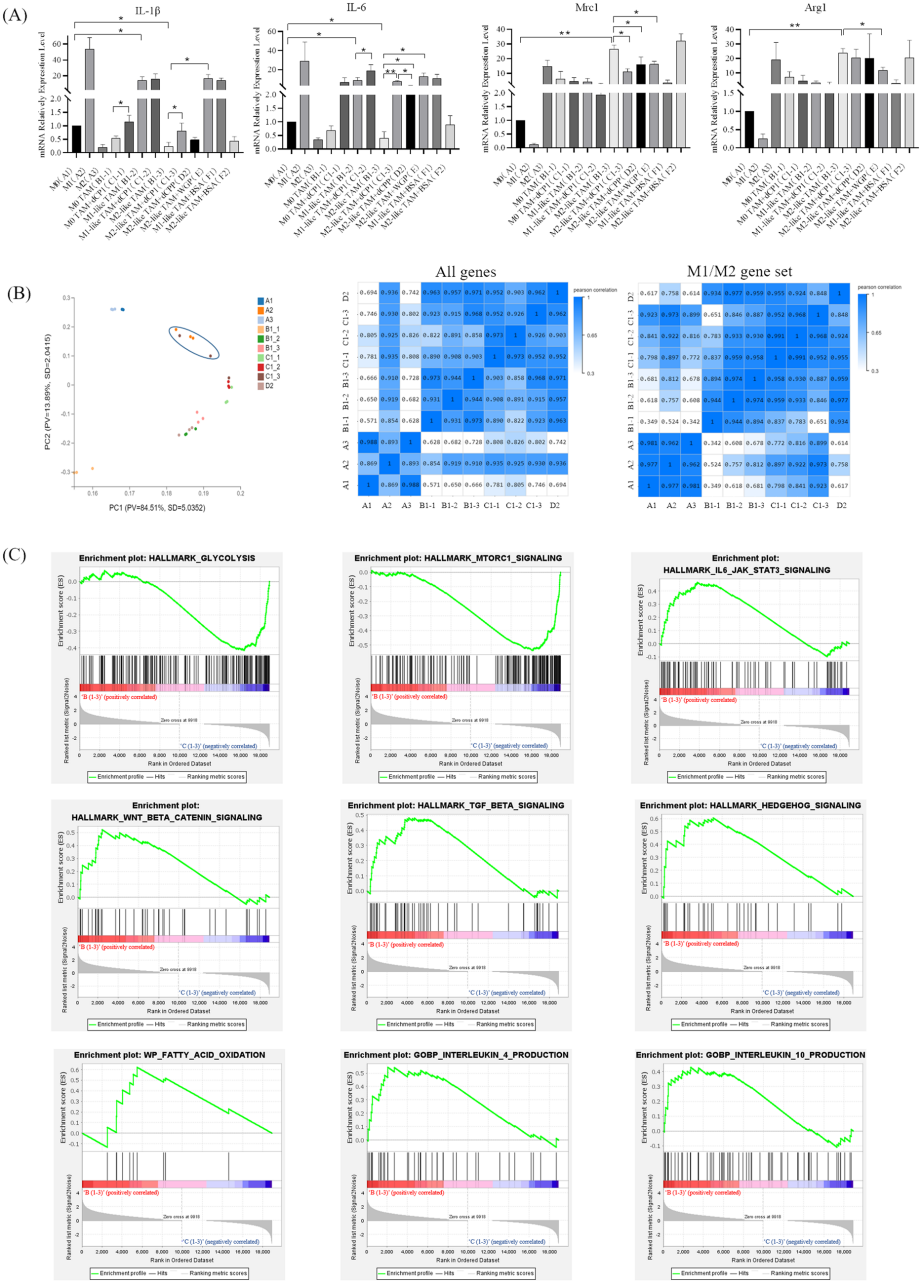
RNA-Seq analysis showed that M2-like TAM after dCP1 treatment tended to be M1 phenotype. (**A**) Effects of polysaccharide dCPP and glycopeptide dCP1 on relative mRNA expression levels of M1/M2 marker genes in M2-like TAMs, *indicates *p* < 0.05, **indicates *p* < 0.01; (**B**) PCA analysis and Pearson's correlation analysis. In PCA analysis, dots represent samples, the same color represents the same sample group, PV represents the Proportion of variance, SD represents standard deviation color represents correlation; in Pearsons correlation analysis, blue represents positive correlation, *P* = 1 represents maximum correlation; (**C**) GSEA analysis of M2-like TAM before and after glycopeptides treatment. *indicates *p* < 0.05, **indicates *p* < 0.01

Firstly, the function of *Codonopsis pilosula* polysaccharide dCPP and glycopeptide dCP1 in repolarization of M2-like TAM phenotype in low levels of glucose, low levels of serum, lipid-rich, and acidic environment was preliminarily analyzed. Some M1 phenotypic marker genes and inflammatory factor genes, and M2 phenotypic marker genes in each group were analyzed by qRT-PCR. As shown in Fig. 2A, compared with macrophages in group A1 without any treatment, the mRNA relative expression levels of IL-1β and IL-6 in group B1-2 were significantly increased, while the mRNA relative expression levels of Mrc1 and Arg1 were also slightly increased, but not statistically significant. The mRNA relative expression levels of IL-1β and IL-6 in group B1-3 were significantly decreased, while the mRNA relative expression levels of Mrc1 and Arg1 were significantly increased (*p* < 0.01). The results show that M1/M2-like TAM modeling is successful in simulated TME.

Compared with untreated M2-like TAM in low glucose, low serum, lipid-rich, and acidic environment (hereafter referred to as M2-like TAM), the relative mRNA expression level of IL-1β in M2-like TAM (group C1-3) was significantly increased after dCP1 treatment. The relative expression level of IL-1β mRNA was significantly increased in M2-like TAM (group E) treated with WGP. WGP is a particulate *Saccharomyces cerevisiae* β-glucan preparation that induces phagocytosis and the production of pro-inflammatory cytokines and reactive oxygen species (ROS) through the activation of Dectin-1 receptors, prompting the conversion of macrophages to the M1 phenotype. In addition, dCP1 can also significantly increase the relative expression level of IL-1β mRNA in TAM in group B1-1. Compared with untreated M1-like TAM (group B1-2), the relative expression level of IL-6 mRNA in M1-like TAM (group C1-2) treated with dCP1 was significantly increased. dCP1, dCPP, and WGP could significantly increase the relative expression level of IL-6 mRNA in M2-like TAM, and compared with M2-like TAM (group D2) treated with dCPP, the relative expression level of IL-6 mRNA in M2-like TAM treated with dCP1 was significantly increased. Compared with M2-like TAM without any intervention, the relative expression level of Mrc1 mRNA in M2-like TAM was decreased after glycopeptide dCP1 (group C1-3), polysaccharide dCPP (group D2), and WGP (group E) treatment, and the decrease was highest after glycopeptide dCP1 treatment. Compared with M2-like TAM without any intervention, the relative expression level of Arg1 mRNA in M2-like TAM was decreased only after WGP treatment.

In conclusion, both *Codonopsis pilosula* polysaccharide dCPP and glycopeptide dCP1 can significantly reduce the relative expression level of Mrc1 mRNA in M2-like TAM in simulated TME, and glycopeptide dCP1 can significantly increase the relative expression level of IL-1β and IL-6 mRNA in M2-like TAM in simulated TME. Moreover, the relative expression level of IL-6 mRNA was significantly increased compared with the polysaccharide dCPP. However, polysaccharide dCPP could only significantly increase the relative expression level of IL-6 mRNA, and the relative expression level was lower than the intervention effect of glycopeptide dCP1 at the same dose. Therefore, we suggest that dCP1 may be better for polarizing M2-like TAM to M1-like TAM compared to dCPP.

### RNA-Seq analysis showed that M2-like TAM was inclined to M1 phenotype after dCP1 treatment

A total of 22,064 effective genes were detected in each group. Dr.Tom system of BGI (Beijing Genomics institution) was used to analyze the data. As can be seen from the results of the principal component analysis (Fig. 2B), the principal component of M2-like TAM treated with dCP1 is more inclined to M1 phenotype macrophages. Further analysis showed that the similarity was more directly displayed by Pearson’s correlation analysis of gene expression matrix. The similarity between C1-3 and A1, A2, and A3 was *P* = 0.746, *P* = 0.930 and *P* = 0.802, respectively. That is, the C1-3 group showed the highest similarity to M1macrophages. Pearson’s correlation analysis was performed on the expression matrix of M1/M2 marker gene of macrophage phenotype. Compared with other groups, M2-like TAM treated with dCP1 showed a higher correlation with M1 macrophages (*P* = 0.973). Compared with C1-3, the principal component of B1-3 was far away from A2 in PCA analysis, and in the M1/M2 marker gene expression matrix, Pearson’s correlation analysis showed that the correlation between B1-3 and M1 macrophages was only *P* = 0.812, far less than that of C1-3 (*P* = 0.973). This indicated that M2-like TAM (group C1-3) was inclined to the M1 phenotype after dCP1 treatment. M1/M2 marker genes are shown in Table S3.

In order to further determine the role of dCP1 in regulating TAM polarization, on the basis of RNA-Seq data, Gene Set Enrichment Analysis (GSEA) was performed on the expression profiles of group B1-3 and group C1-3 using GSEA4.3.1 software. Both the software and gene set were obtained from the MSigDB database website [48–50]. We mainly compared the changes of representative gene sets related to phenotype and function of M2-like TAM before and after glycopeptides dCP1 treatment (Fig. 2C). By comparison, it was found that after dCP1 treatment, the gene set Hallmark Glycolysis (NES = −1.3982755, *p* < 0.001) related to glycolysis was up-regulated, and the energy supply of M1 macrophages mainly depended on glycolysis as a fast energy source. The energy supply mode of M2-phenotypic macrophages is mainly oxidative phosphorylation [51]. In addition, the Hallmark MTORC1 Signaling gene set (NES = −1.4287497, *p* < 0.001) was up-regulated, and mTORC1 up-regulation was found to promote glycolysis [52]. Compared with the glycopeptides-treated groups, Hallmark IL-6-KAK-Stat3 Signaling (NES = 1.3829753, *p* < 0.001), Hallmark Wnt Beta Catein Signaling (NES = 1.3512646, p = 0.1870229), Hallmark TGF Beta Signaling (NES = 1.2529285, *p* = 0.08349146), and Hallmark Hedgehog Signaling (NES = 1.3689349, *p* < 0.001) and other gene sets were up-regulated in control groups. In addition to the above gene set, GOBP Interleukin-4 Production (NES = 1.2952715, *p* = 0.10144927) and GOBP Interleukin-10 Production (NES = 1.2340547, *p* = 0.1904762), two kinds of anti-inflammatory factor production gene sets were also up-regulated in control groups.

In general, the gene sets of various signaling pathways that promote the polarization of macrophages toward the M2 phenotype were up-regulated before glycopeptides dCP1 treatment, but these gene sets were not up-regulated after dCP1 treatment, indicating that the M2 phenotype of TAM was inhibited. In addition, after dCP1 treatment, the glycolysis gene set of TAM was up-regulated, indicating that its metabolism and energy supply pattern shifted to M1 phenotype. According to the above analysis, M2-like TAM tended to be M1 phenotype after being treated with glycopeptides dCP1.

### PI3K-AKT pathway may have the most important effect in dCP1 regulation of M2-like TAM polarization toward M1 phenotype

**Fig. 3:**
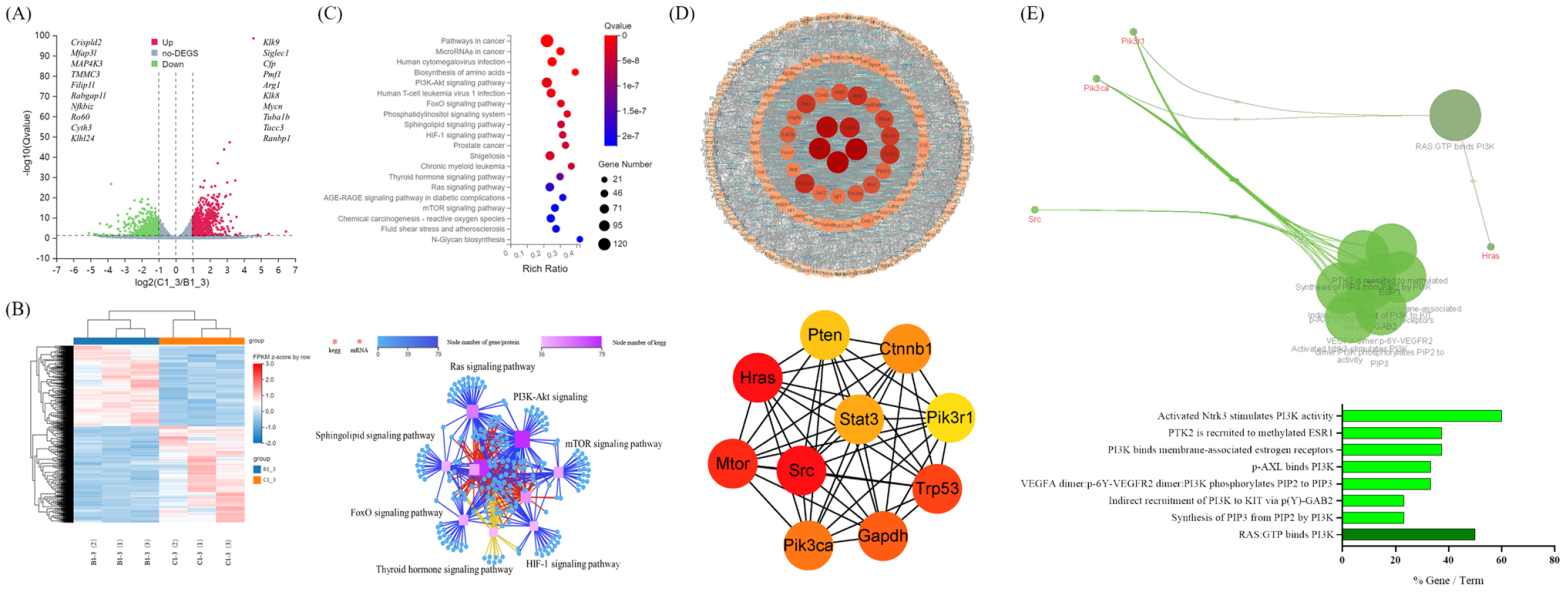
Differential gene analysis and screening and analysis with Hub genes. (**A**) Volcano plot of differential genes; red represents up-regulated genes and green represents down-regulated genes; (**B**) Heat map of differential gene clustering. Genes are represented horizontally, one sample per column; red indicates highly expressed genes and blue indicates low expressed genes; (**C**) Enrichment of the KEGG pathways associated with macrophage polarization and its relational network. Different node shapes in the network diagram indicate different contents, squares indicate KEGG Pathway, and circles indicate mRNA. Both the color and size indicate the number of genes or transcripts connected to the node. The darker the color and larger the square, the more genes or transcripts are connected to the node. The line indicates the relationship between two signaling pathways, and the more the number of upstream and downstream signaling pathways, the more important they are in the network. Different color lines indicate different classifications of pathway, red indicates cellular processes, blue indicates environmental information processing, green indicates genetic information processing, purple indicates human diseases (animals only), orange indicates metabolism, yellow indicates organic systems, and brown indicates drug development; (**D**) PPI analysis and Hub gene screening; (**E**) reactome reaction analysis of Hub genes

RNA-Seq data analysis showed that 3366 genes were differentially expressed between group B1-3 and group C1-3. It can be seen from the volcano map of DEGs (Fig. 3A) that there are 1,836 up-regulated genes and 1,530 down-regulated genes, and the distribution of differentially expressed genes on the left and right sides of the volcano map is basically balanced. The top 10 genes with the highest significance among the up-regulated and down-regulated genes were labeled, and among the 10 genes with the most significant up-regulated, the expression products of Siglec-1 [53], *KLK9* [54], *CFP* [55], *PMFI* [56], *KLk8* [57] and *TUBA1B* [58] have pro-inflammatory effects. Among the 10 most significantly down-regulated genes, *Crispld2* [59–61], *MAP4K3* [62] and *Nfkbiz* [63] had anti-inflammatory effects. In the heatmap, genes are represented horizontally, with one sample in each column (Fig. 3B). Red represents genes with high expression and blue represents genes with low expression. The B1-3 and C1-3 differential genes were classified by KEGG Pathway Level 2, and the pathways related to metabolic reprogramming, macrophage polarization, and immune system were selected for KEGG Pathway enrichment. The pathways related to metabolic reprogramming and polarization of macrophages (*q* valve < 0.05) were screened for further analysis (Fig. 3C). The pathways are biosynthesis of amino acids, PI3K-Akt signaling pathway, FoxO signaling pathway, phosphatidylinositol signaling system, sphingolipid signaling pathway, HIF-1 signaling pathway, thyroid hormone signaling pathway, Ras signaling pathway, mTOR signaling pathway, N-Glycan biosynthesis.

The analysis of the pathway network map showed that the PI3K-AKT pathway map obtained by KEGG classification and KEGG enrichment method had the most linked genes, and the number of upstream and downstream signaling pathways was the largest, indicating that it was the most important pathway (Fig. 3C). After dCP1 treatment, in the PI3K-AKT pathway, the gene expression of its core pathway changed. As shown in Fig. S1A, the expression of *Pik3ca*, *Pik3r1*, *Pik3r3* and *Pik3cb* was reduced. Downstream of the PI3K-AKT pathway, the expression of Brca1-, Gys1-, Pck2-, and Trp53-related macrophage polarization genes was up-regulated, while the expression of Myc and Mcl1 was downregulated.

In addition to the above KEGG pathway enrichment analysis, select the top 100 genes with the highest significance among the up-/down-regulated DEGs for KEGG Module analysis, and it was found the significantly up-regulated genes were mainly in glycolysis, C1 unit interconversion, and pyrimidine deoxyribonucleotide biosynthesis modules (Fig. S1B), while the significantly down-regulated genes were mainly in lipid metabolism-related modules such as sphingolipids (GSLs) biosynthesis and phosphatidylethanolamine (PE) biosynthesis (Fig. S1C).

### Analysis of the hub genes related to M2-like TAM repolarization found that the significant reaction responses or pathways were related to PI3K and PI3K-AKT pathways

To find out the Hub genes about M2-like TAM repolarization in the DEGs identified in RNA-Seq, PPI analysis and Hub gene screening were performed on the DEGs in the pathways associated with macrophage metabolic reprogramming and polarization after enrichment by KEGG Pathway using STRING [64] and Cytoscape 3.9.1 and analyzed by Degree algorithm, and ranked (Fig. 3D). Hub genes are listed in Supplementary Table S4. These indicated that the above genes acted as connection hubs in the process of dCP1-mediated polarization from M2-like TAMs to M1-like TAMs. Use ClueGo [65] to perform Reaction analysis on Hub genes, set Network specificity to medium, the minimum number of genes in Term/Pathway selection is 3%, and the proportion is greater than 20%, and only significant reactions or pathways with *p* < 0.05 are displayed. Through the analysis, it was found that the significant reactions or pathways were all related to the PIK3 and PI3K-AKT pathways (Fig. 3E), which coincided with the above-mentioned KEGG network analysis that found that this pathway was the most important.

### Global analysis of lipid metabolites revealed that the optimal characteristic subnetwork of M2-like TAM before and after dCP1 treatment was the conversion between LPC and PC

A total of 523 lipid metabolites meeting the requirements were detected in all experimental groups, typical base peak chromatograms for each group of samples visually demonstrate the detection of metabolites in the samples (Fig. S2 A, B), and the number of metabolites was counted according to Sub Class (Fig. S2C). The PCA model between all experimental groups was developed in order to analyze the distribution and separation trend of each experimental group (Fig. S2D). The analysis shows that in this model, the first principal component accounts for 62.07% of the total variance and the second principal component accounts for 17.42% of the total variance. The differences between groups in each experimental group were large and could be completely separated. Among them, the differences between B1-3 (control group, M2-like TAM without dCP1 treatment) and C1-3 (M2-like TAM with dCP1 treatment) groups were larger before and after glycopeptide treatment, while the differences between B1-2 and C1-2 groups were smaller. Therefore, the subsequent study only analyzed B1-3 and C1-3. In addition to this, the degree of aggregation of samples within each experimental group was high, the intra-group differences were small, and there were no abnormal samples.

**Fig. 4:**
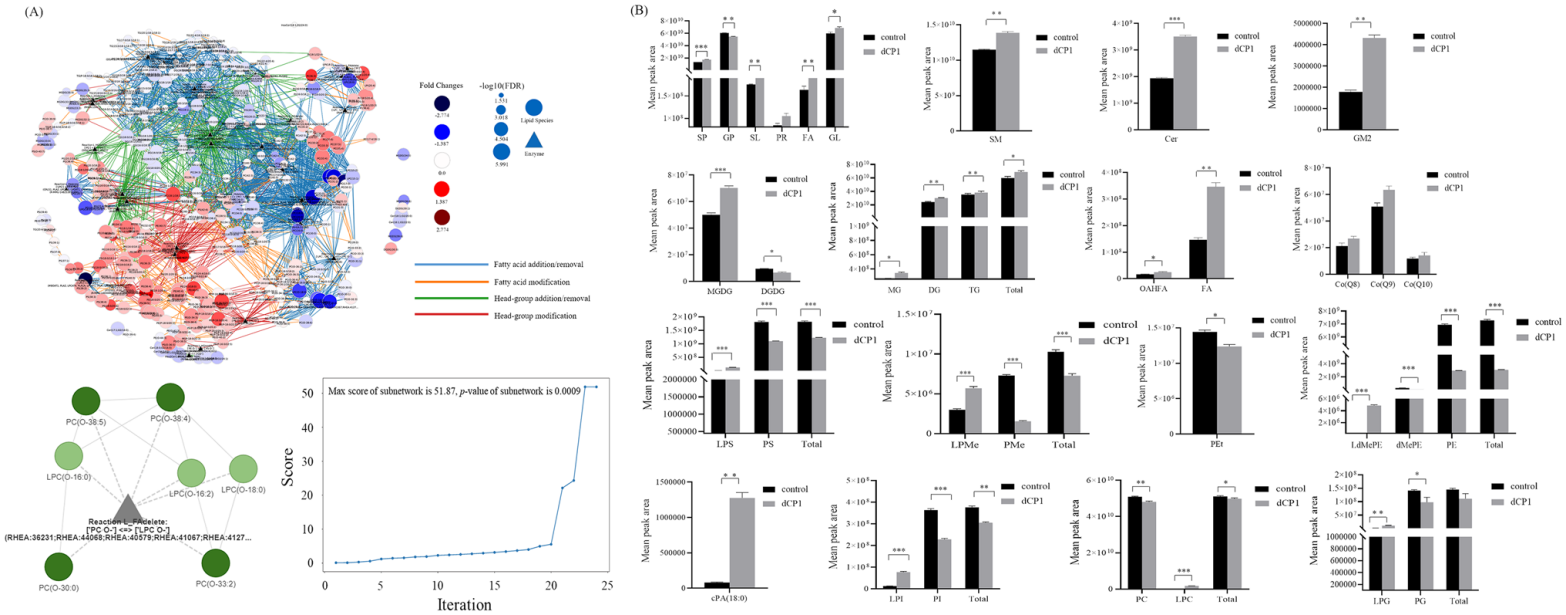
Global analysis and total relative content analysis of lipid metabolites in the control and dCP1-treated groups. (**A**) Super-network of lipid reactions with enzyme involvement, optimal sub-networks, and score changes during algorithm optimization. The circular nodes in the figure represent various lipids, and the red color of the lipid nodes represents the lipids with higher content in the control group compared to those with higher content after dCP1 treatment in blue. Node size is proportional to the -log10(FDR) value. The color of the connecting line indicates the type of reaction connecting the two nodes, where blue indicates fatty acid addition or removal, orange identifies fatty acid modification, green indicates head group addition or removal, and red indicates head group modification; (**B**) Relative content analysis of main classes and subclasses of lipid metabolites. *indicates *p* < 0.05, **indicates *p* < 0.01, ***indicates *p* < 0.001

The LINEX2 online tool [66] was used to conduct a global analysis of lipid metabolites in the control group and glycopeptides dCP1-treated group. Figure 4A shows the lipid reaction supernetwork involving enzymes in M2-like TAMs before and after treatment with glycopeptides dCP1, important characteristic subnetworks, and score changes during algorithm optimization. The circular nodes in the supernetwork represent various lipids, the red of the lipid node represents the lipid with higher content in the control group, and the blue represents the lipid with higher content after dCP1 treatment. Node size is proportional to the −log10(FDR) value. The color of the connecting line indicates the type of reaction connecting two nodes, where blue indicates the addition or removal of fatty acids, orange indicates the modification of fatty acids, green indicates the addition or removal of headgroups, and red indicates the modification of headgroups. Figure 4A shows that in the red lipid accumulation area, that is, in the control group, the head group modification reaction and a part of the fatty acid modification reaction are mainly carried out. In the treatment group, lipids mainly underwent fatty acid and head group addition or removal reactions. Afterward, the lipid network is enriched, and the important characteristic subnetworks of M2-like TAM after glycopeptides dCP1 treatment are found by using a local search supported by simulated annealing. The principle is that local search optimization is performed by applying local changes to candidate solutions. Study the search space so that the objective function value increases. These changes will be applied until no more local improvements can be made. To avoid getting stuck at local maxima, the simulated annealing process [67] allows non-optimal solutions, thereby increasing the exploration space. The best subnetwork is returned when no further improvement is possible or the condition is no longer satisfied with simulated annealing or when the maximum number of iterations is reached. The figure shows that the optimal subnetwork is composed of PC, LPC lipids, and corresponding reactions, which represent the conversion between LPC and PC, and the detailed reactions are shown in Supplementary Table S5.

### Analysis of the relative content of lipid metabolites showed that the lipid metabolites profile of M2-like TAM after dCP1 treatment was similar to M1 macrophages

After analyzing the lipid metabolites of the dCP1 glycopeptide treatment group and the control group, it was found that after dCP1 treatment, the sphingolipids (Sphingolipids, SP) and glycolipids (Saccharolipids, SL), the total relative content of fatty acyls (Fatty Acyls, FA) and glycerolipids (Glycerolipids, GL) metabolites increased significantly, the total relative content of glycerophospholipids (GP) metabolites decreased significantly, and although the total relative content of Prenol Lipids (PR) metabolites increased, there was no significant change (Fig. 4B).

Compared with the control group, after dCP1 glycopeptide treatment, the total relative content of sphingolipid metabolites in M2-like TAM was significantly increased, including the total relative content of sphingolipids, ceramide (Cer), and ganglioside GM2. The total relative content of neutral glycerides in glyceride metabolites increased significantly, among which the total relative contents of monoglyceride (MG), diglyceride (DG), and triglyceride (TG) all increased significantly. The total relative content of saccharolipids metabolites increased, among which the total relative expression of monogalactosyldiglyceride (MGDG) increased significantly, while the total relative expression of digalactosyldiglyceride (DGDG) decreased significantly. The total relative content of fatty acid metabolites increased significantly, among which the total relative content of fatty acid (FA) and (O-acyl)-1-hydroxy fatty acid (OAHFA) all increased significantly. There is an increase in the total relative content of coenzyme (Co) class of prenol lipids metabolites, in which the relative content of CoQ8 and CoQ10 decreased and the relative content of CoQ9 increased, but none of the changes were significant. The relative content of P-Ethanol Amine (P-Eth) metabolites in the GP class of metabolites decreased, with a significant increase in the total relative content of lyso-dimethylphosphatidylethanolamine (LdMePE) and a significant decrease in the total relative content of both dimethylphosphatidylethanolamine (dMePE) and phosphatidylethanolamine (PE). There is a significant decrease in the total relative content of phosphatidylserine (P-Serine) metabolites, with a significant increase in the total relative content of lyso-phosphatidylserine (LPS) and a significant decrease in the total relative content of phosphatidylserine (PS). There is a significant decrease in the total relative content of phosphatidylmethanol (P-Methanol) metabolites, with a significant increase in the total relative content of lyso-phosphatidylmethanol (LPMe) and a significant decrease in the total relative content of phosphatidylmethanol (PMe). There are a significant increase in the relative content of cyclophosphatidic acid (cPA) and a significant decrease in the total relative content of phosphatidylethanol (PEt). Total relative content of phosphatidylglycerol (P-Glycerol) metabolites decreased, with a significant increase in total relative content of lyso-phosphatidylglycerol (LPG) and a significant decrease in total relative content of phosphatidylglycerol (PG). There is an increase in the total relative content of phosphatidylinositol (P-Inositol) metabolites, with a significant increase in the total relative content of phosphatidylinositol (PI) and a significant decrease in the total relative content of lyso-phosphatidylinositol (LPI). There is a decrease in the total relative content of phosphatidylcholine (P-Choline) metabolites, with an increase in the total relative content of lysophosphatidylcholine (LPC) and a decrease in the total relative content of phosphatidylcholine (PC). According to the data above, there was an increase in the relative contents of lipid metabolites like SM, Cer, GM2, TG, DG, MG, LPS, LPI, and LPC, while a decrease was seen in the relative contents of lipid metabolites like PS, PEt, PC, and PG. There was still a minor amount of lipid metabolism with M2 phenotype characteristics after horizontal comparison, but the majority of the expression characteristics of the lipid profile of TAM treated with dCP1 glycopeptide were comparable with the M1 phenotype lipid metabolism profile [68–70]. Figure S3 displays more specific modifications in lipid metabolism.

### The interconversion between PE and PC, as well as PC and DG are core reactions of key differential metabolic

**Fig. 5:**
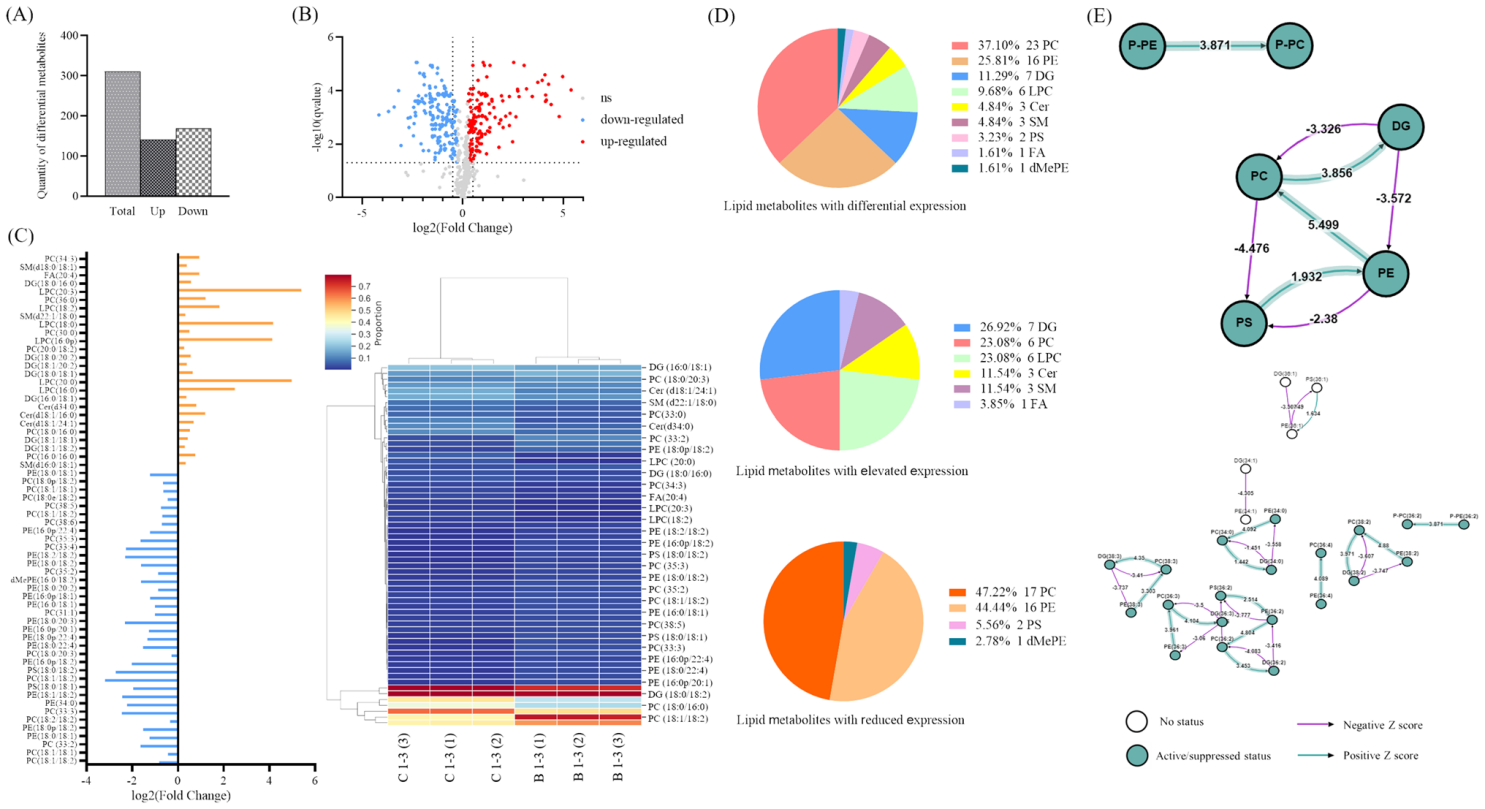
Screening and analysis of key differential lipid metabolites. (**A**) Differential metabolite statistics; (**B**) Differential metabolite volcano map; (**C**) Relative expression of key differential lipid metabolites with VIP value > 1; (**D**) Classification of key differential lipid metabolites with VIP value > 1; (**E**) Key differential lipid metabolites reaction analysis of subclasses and specific lipid molecules

The analysis of Fold Change values and q value values of various lipid metabolites in the dCP1-treated group compared to the control group revealed that M2-like TAM had a total of 310 differential metabolites after dCP1 treatment, including 141 up-regulated metabolites and 169 down-regulated metabolites (Fig. 5A). The differential metabolite volcano plot is shown in Fig. 5B.

After univariate analysis, a total of 310 lipid metabolites were obtained, which is a large number and not conducive to the screening of key lipid metabolism changes. Therefore, based on this, the OPLS-DA model was used for further screening of 310 lipid metabolites to obtain key differential lipid metabolites. The OPLS-DA model was built and analyzed by SIMCA software. From Figure S4A, it can be seen that there is a clear difference between the two groups. The fit index of the independent variable (R2x) in this analysis is 0.995, the fit index of the dependent variable (R2y) is 1.000, the model prediction index (Q2) is 1.000, and R2 and Q2 over 0.5 indicate acceptable model fit results. After 200 permutation tests, as shown in Figure S4B, the regression lines show that the Q values in the stochastic model are smaller than those in the original model, and the intersection point of the Q2 regression line with the vertical axis is less than zero, indicating that the model is not over-fitted and the model validation is effective. In summary, all results indicate high model reliability. The relative expression of key differential lipids in the control and dCP1-treated groups is shown in the heatmap in Fig. 5C. (The data were normalized by min–max and analyzed using Heml 2.0.), the types and percentages of key differential metabolites are shown in Fig. 5D. The analysis found that, compared with the control group, after dCP1 treatment, among the 62 key differential lipid metabolites with VIP > 1 in M2-like TAMs, 26 were up-regulated and 36 were down-regulated. The specific information on the 62 key differential lipid metabolites is shown in Table S6.

BioPAN (Bioinformatics Methodology for Pathway Analysis) [71, 72] was used to analyze the screened key differential metabolites. BioPAN can explore systematic changes in lipid reaction pathways at the level of lipid subclasses and lipid molecule species in different experimental groups, and changes in gene activity can also be predicted. BioPAN calculates a Z-score for each weighted pathway or reaction to determine its status. A t-test between treatment and control groups is used to generate *p* values, which can be converted to Z-scores by *Z* = CDF−1(1−*p*), where CDF is the cumulative distribution function. *Z* values > 1.645 are considered active (suppressed) responses, and *Z* values < 1.645 are considered inactive (non-suppressed) responses. Compared with the control group, in the reactions carried out at the lipid subclass level in the M2-like TAM treated with dCP1 (Fig. 5E), the PE → PC → DG, PS → PE → PC → DG, P-PE → P-PC, and PC → DG reaction chains were in the active state (Table 1); the reaction chains consisting of DG → PE → PS, DG → PC, PE → PS, and PC → PS → PE reaction chains are in the inhibited state (Table 2), and the reactions at the level of specific lipid molecules are shown in Tables S7 and S8, and the interconversion between phosphatidyl ethanolamine (PE) and phosphatidyl cholines (PC) metabolites, as well as between PC and diacyl glycerol (DG) metabolites, may be the core reactions of glycopeptide dCP1 regulating the transformation of M2-like TAM to M1 phenotype.

**Table 1:** Lipid subclass active reaction (dCP1 treatment vs control)

Reaction chain	Z-score	Predicted genes
PE → PC → DG	6.615	PEMT
PS → PE → PC → DG	6.517	PISD, PEMT
P-PE → P-PC	3.871	CEPT1, PLD1, PLPP1, PLPP2, CHPT1
PC → DG	3.856	–

**Table 2:** Lipid subclass suppressed reaction (dCP1 treatment vs control)

Reaction chain	Z-score	Predicted genes
DG → PE → PS	4.209	CEPT1, PTDSS2
DG → PC	3.326	CHPT1
PE → PS	2.38	PTDSS2
PC → PS → PE	1.799	PTDSS1, PISD

### Molecular docking and molecular dynamics simulations reveal that the glycopeptide in dCP1 may exert phenotypic regulation by binding to TLR2 on TAM

The above studies revealed that the glycopeptide dCP1 has the ability to regulate the polarization of M2-like TAM to M1 phenotype, and the function of many active substances such as glycoproteins/glycopeptides is determined by their structure, so their structure was characterized and the relationship between structure and function was explored. The scores were ranked, and the top 9 glycopeptides with highest confidence were selected, namely NLGS*VAGPR (Score = 259.09), QHYRS*R (Score = 251.13), AVGRGLVS*SCICVGR (Score = 239.75), GYGASAQAALVT*R (Score = 212.52), ELGFIS*KAPR (Score = 206.54), IVALT*NAK (Score = 173.94), GT*QDLFLAR (Score = 172.59), VQGLLPS*MVK (Score = 172.55) and ITN*K (Score = 170.36), a total of eight O-glycosylated peptides and one N-glycosylated peptides. The above glycopeptides all contained basic amino acids or hydrophobic amino acids, indicating that these glycopeptides have the structural basis to become functional peptides. Therefore, the above glycopeptides were selected for the next step of analysis. The extracted ion flow diagram, MS spectrum, and MS/MS spectrum of the glycopeptides are shown in Figure S5. Based on the glycopeptides LC–MS/MS data to obtain the amino acid sequence, carbohydrate chain composition, and glycosylation information of each glycopeptide, the glycopeptides’ structures were modeled to obtain the 3D structural data pdb files of the glycopeptides for further study of glycopeptides–receptor interactions. The conformations of the nine glycopeptides are shown in Figure S6. Molecular docking is used to determine which receptors the glycopeptides interact with and thus attempt to elucidate the mechanism by which it regulates TAM polarization.

The receptors expressed in macrophages that recognize and bind to glycoproteins or glycopeptides are mainly Toll-like receptor 2, NOD1, and part of the C-type lectin superfamily, such as Complement component C1q receptor, C-type lectin domain family 4 member A, C-type lectin domain family 4 member D, C-type lectin domain family 4 member E, C-type lectin domain family 5 member A, C-type lectin domain family 7member A, C-type lectin domain family 10 member A, C-type lectin domain family 12 member A, CD209 antigen-like protein A, CD209 antigen-like protein B, CD209 antigen-like protein D, and mannose-binding protein A, macropage mannose receptor 1, oxidized low-density lipoprotein receptor 1, tetranectin, etc. Therefore, the above receptors were selected as potential receptors for glycopeptides dCP1, and their pdb files containing 3D structures were obtained from the RCSB database and AlphaFold Protein Structure Database. The pdb code numbers and conformations of the receptors are shown in Figure S7.

Molecular docking of 9 glycopeptides with various selected receptors was performed using ZDOCK 3.0.2. The ZDOCK program evaluates each binding model mainly by searching all possible binding modes in the space obtained by translation and rotation between receptor and ligand, using a comprehensive scoring function based on IFACE statistical potentials, structural complementarity, and electrostatic composition. The docking scores of each glycopeptide with the receptor are shown in Figure S8. After docking, it was found that all 9 glycopeptides had the highest docking scores with TLR2 receptor molecules and were much higher than other receptors, with GT(DGalpNAcα1-3DGalpNAcα1-OH)QDLFLAR having the highest score of 2013.616; QHYRS(DGalpα1- 3DGalpNAcα1-OH)R had the lowest score of 1378.462. It is inferred that the glycopeptides dCP1 may be recognized and bound mainly by TLR2.

**Fig. 6:**
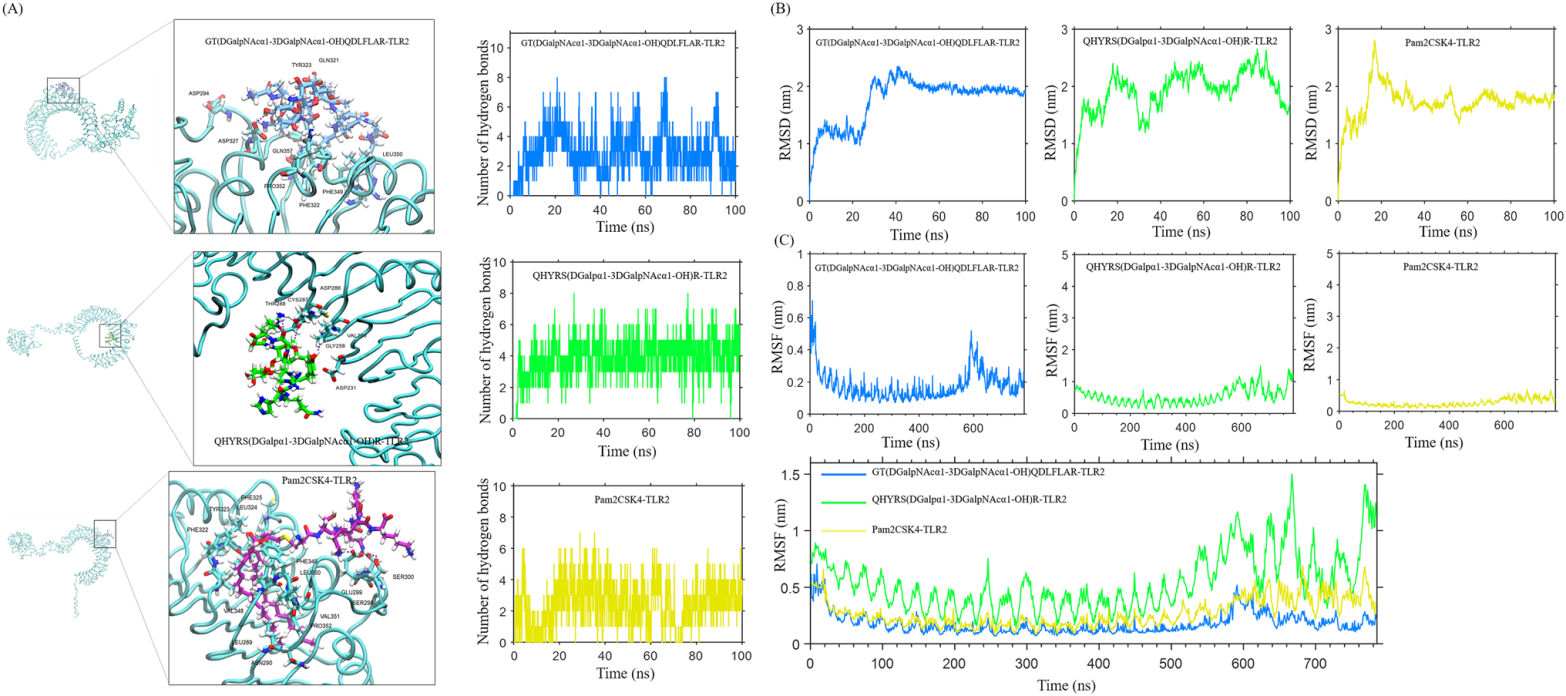
Molecular dynamics analysis of glycopeptide and TLR2 docking complexes. (**A**) RMSD analysis of the complex; (**B**) RMSF analysis of the complex; (**C**) Conformation and number of hydrogen bonds of the complex

The glycopeptides GT(DGalpNAcα1-3DGalpNAcα1-OH)QDLFLAR with the highest docking score to TLR2 and the glycopeptides QHYRS(DGalpα1-3DGalpNAcα1-OH)R with the lowest score were selected for molecular dynamics simulations. The TLR2 agonist lipopeptide Pam2CSK4 was selected as a control [73]. Three complex GT(DGalpNAcα1-3DGalpNAcα1-OH)QDLFLAR-TLR2, QHYRS(DGalpα1-3DGalpNAcα1-OH)R-TLR2 and Pam2CSK4-TLR2 were obtained after docking as the initial conformation, and 100 ns molecular using AMBER 14 force field dynamics simulations were performed to obtain conformations and trajectories, and data were compiled and analyzed for the conformations and trajectories. The conformation was analyzed at the lowest free energy state, GT(DGalpNAcα1-3DGalpNAcα1-OH)QDLFLAR interacts with ASP327 (distance 3.2 Å) and GLN357 (distance 2.1 Å) in TLR2 receptor protein through hydrogen bonding, and with ASP294, GLN321, PHE322, TYR323, PHE349, LEU350, and PRO352 through hydrophobic interactions (Fig. 6A); QHYRS(DGalpα1-3DGalpNAcα1-OH)R interacts with TLR2 receptor proteins in GLY258 (distance 2.5 Å), ASP286 (distance 2.1 Å), CYS287 (distance 2.2 Å), and THR288 (distance 2.5 Å) through hydrogen bonding and interacts with ASP231 and VAL260 through hydrophobic interactions (Fig. 6A); Pam2CSK4 interacts with GLU299 (distance 2.3 Å) and SER300 (distance 2.2 Å) in TLR2 receptor protein through hydrogen bonding and interacts with SER298, PHE322, TYR323, LEU324, PHE325, ASN290, LEU289, VAL348, PHE349, LEU350, VAL351, and PRO352 through hydrophobic interactions (Fig. 6A). The hydrophobic interaction of Pam2CSK4 was significantly enhanced compared to the other glycopeptides, attributed to the hydrophobic lipid chains of the lipopeptides.

From the specific amino acid sites of action, it can be seen that GT(DGalpNAcα1-3DGalpNAcα1-OH)QDLFLAR can form hydrophobic pockets with both Pam2CSK4, which can better stabilize the protein, that means better bind to TLR2; QHYRS(DGalpα1-3DGalpNAcα1-OH)R cannot form hydrophobic pockets with the protein hydrophobic pocket and therefore has a weaker effect. The number of hydrogen bonds between TLR2 and GT(DGalpNAcα1-3DGalpNAcα1-OH)QDLFLAR was continuously stabilized with the spatial configuration throughout the simulation, during which the number of hydrogen bonds increased from 0 and finally stabilized at a mean value of all around 2; the number of hydrogen bonds between TLR2 and QHYRS(DGalpα1-3DGalpNAcα1-OH)R is continuously stabilized with the spatial configuration throughout the simulation, and finally stabilized at the mean value of about 4; the number of hydrogen bonds between TLR2 and Pam2CSK4 was continuously stabilized with the spatial configuration throughout the simulation, during which the number of hydrogen bonds increased from 0 and finally stabilized at the mean value of both around 2 (Fig. 6A).

Afterward, the root mean square deviation (RMSD) values were calculated from the trajectory analysis to obtain images of the variation in the RMSD values with time (Fig. 6B). The GT(DGalpNAcα1-3DGalpNAcα1-OH)QDLFLAR-TLR2 complex reached equilibrium after about 60 ns, and the Pam2CSK4-TLR2 complex reached equilibrium after about 40 ns. The average RMSD values of both were stable at around 20 Å, which was within the acceptable range, indicating that both ligands could bind tightly to the TLR2 receptor. In contrast, QHYRS(DGalpα1-3DGalpNAcα1-OH)R did not reach equilibrium within the simulated time, and the overall fluctuations during the simulation were large, indicating that the compound was relatively unstable in binding to the receptor. The root means square perturbation (RMSF) measures the fluctuating distance of each amino acid residue relative to the equilibrium position during the simulation, and the height of the peak describes the fluctuation of the corresponding amino acid residue. From Fig. 6C, it can be seen that in the GT(DGalpNAcα1-3DGalpNAcα1-OH)QDLFLAR interaction with TLR2, the protein fluctuates more around the head, amino acid 600; in the Pam2CSK4 interaction with TLR2, the overall fluctuation of the protein is smaller, which also indicates that the binding of Pam2CSK4 to TLR2 protein stable, while QHYRS(DGalpα1-3DGalpNAcα1-OH)R fluctuated more with TLR2 protein binding. The above results suggest that both GT(DGalpNAcα1-3DGalpNAcα1-OH)QDLFLAR and Pam2CSK4 can stabilize this region protein well. In addition to this, it can also be seen that the RMSF values of the key residues of GT(DGalpNAcα1-3DGalpNAcα1-OH)QDLFLAR and Pam2CSK4 interacting with TLR2 are at a relatively low level with an average value of about 10 Å, indicating that the key residues are inflexible due to the binding of the TLR2 receptor to the two glycopeptides ligands and the binding is very stable. The above results indicate that both GT(DGalpNAcα1-3DGalpNAcα1-OH)QDLFLAR and Pam2CSK4 can bind well to TLR2 protein.

The binding free energy between ligand and receptor was calculated by the Molecular Mechanics/Poisson Boltzmann Surface Area (MM/PBSA) method. The equation to calculate the binding free energy as follow:

$$\Delta E_{bind} = \, \Delta E_{vdw} + \Delta_{Eele} + \Delta E_{solv} + \Delta E_{SASA}$$

The binding stability of the TLR2 receptor complexes after docking with glycopeptides was assessed by calculating the binding free energy of the complexes. Molecular dynamics simulations were calculated for the three complexes and the lower the binding free energy, the higher the binding stability was indicated. The contribution of each energy to the binding free energy is shown in Table 3, and the results show that the main sources of both ligand and substrate ΔEbind are ΔEvdw and ΔEele. ΔEsolv, as the only source of resistance for ΔEbind, can be offset by ΔEvdw and ΔEele. For the GT(DGalpNAcα1-3DGalpNAcα1-OH)QDLFLAR-TLR2 complex, the binding free energy was −199.712 ± 34.569 kJ/mol; for the QHYRS(DGalpα1-3DGalpNAcα1-OH)R-TLR2 complex, the binding free energy was −44.419 ± 27.775 kJ/mol; for the Pam2CSK4-TLR2 complex, the binding free energy was −363.335 ± 37.058 kJ/mol.

**Table 3:** Analysis of binding free energy

	ΔEvdw	ΔEele	ΔEsolv	ΔESASA	ΔEbind
GT(DGalpNAcα1-3DGalpNAcα1-OH)QDLFLAR-TLR2	−222.084 ± 21.999	−249.386 ± 40.338	306.731 ± 53.715	−34.703 ± 2.030	−199.712 ± 34.569
QHYRS(DGalpα1-3DGalpNAcα1-OH)R-TLR2	−99.869 ± 26.705	−214.399 ± 44.150	286.135 ± 60.320	−16.293 ± 3.250	−44.419 ± 27.775
Pam2CSK4-TLR2	−368.782 ± 24.276	−521.966 ± 139.283	583.102 ± 160.892	−55.689 ± 3.477	−363.335 ± 37.058

By comparison, the binding capacity of GT(DGalpNAcα1-3DGalpNAcα1-OH)QDLFLAR to TLR2 was found to be higher than that of QHYRS(DGalpα1-3DGalpNAcα1-OH)R to TLR2, which is consistent with the results obtained by molecular docking. Although weaker than the binding ability of Pam2CSK4 to TLR2, GT(DGalpNAcα1-3DGalpNAcα1-OH)QDLFLAR is also susceptible to TLR2 binding, considering the immunomodulatory function of dCP1 we found, it may act as a TLR2 agonist to promote the polarization of M2-like TAM to M1 phenotype.

## Discussion

To examine whether the glycopeptide in the *Codonopsis pilosula* extracts has the ability to polarize M2-like TAM to M1 phenotype, we first purified the glycopeptide dCP1 from dCPCP and found that both dCP1 and dCPP could regulate M2-like TAM to M1 phenotype polarization in the preliminary TAM model. To make the study close to the real physiological environment, we designed a TAM model in the simulated tumor microenvironment. Nutritional deficiency is one of the hallmark conditions of the tumor microenvironment. Due to the abnormal angiogenesis and insufficient blood supply in tumor tissues, the rapid growth of tumors leads to the formation of a hypoxic and nutrient-deficient microenvironment within the core of tumors. In TME, tumor cells use aerobic glycolysis for energy supply, which results in large amounts of CO_2_ and lactate ions being produced in the tumor and excreted to the extracellular compartment, resulting in an acidic tumor microenvironment [74–77]. In addition to this, TME is also rich in fatty acids, and various solid tumors have been reported to secrete and accumulate more fatty acids, resulting in a fatty acid-rich tumor microenvironment. This imbalance of fatty acids and lipids accumulated within TME also leads to metabolic changes in tumor-infiltrating immune cells [78–83]. So, all cells in a tumor, including immune cells, are in the low levels of glucose, low levels of oxygen, acidic, lipid-rich tumor microenvironment.

The TAM model in the simulated tumor microenvironment was treated with dCP1 and dCPP, respectively, and it was found that glycopeptide can better reduce Mrc1 in M2-like TAM and increase the relative expression levels of IL-1β and IL-6 mRNA. Therefore, it is believed that glycopeptide dCP1 has a better effect. In order to fully explore the effect of dCP1 on TAM polarization, RNA-Seq was used to study the effect of dCP1 on TAM polarization at the transcriptional level. The results showed that the principal components of M2-like TAMs treated with dCP1 were more inclined to M1-type macrophages, and the Pearson’s correlation analysis and GSEA analysis of the gene expression matrix showed this similarity more intuitively, that is, M2-like TAM treated with dCP1 tended to M1 phenotype at the transcriptional level.

GSEA revealed that the gene set Hallmark Glycolysis as well as Hallmark mTORC1 Signaling, which are related to glycolysis, was upregulated after treatment with the glycopeptides dCP1 compared to controls. M1 macrophages are able to sustain inflammatory responses and kill pathogens, relying mainly on aerobic glycolysis and fatty acid biosynthesis, and mTORC1 upregulation promotes glycolysis to proceed [52]. This metabolic adaptation facilitates the rapid production of ATP to maintain its phagocytic function [84] and provides metabolic precursors to feed the pentose phosphate (PPP) pathway. And its tricarboxylic acid cycle (TCA) is divided into two parts to provide precursors required for the synthesis of several lipids as well as to stabilize transcription factors [85], such as HIF1α, which have a key role in activating glycolysis, while fatty acids are precursors for the synthesis of inflammatory mediators [86]. Compared to the glycopeptide dCP1 treated group, Fatty acid beta oxidation, WP fatty acid oxidation, Hallmark IL-6-JAK-STAT3 signal transduction, Hallmark Wnt β serial signal transduction, Hallmark TGF β signal transduction, and Hallmark Hedgehog Signal transduction were upregulated in the control. Fatty acid beta-oxidation is one of the metabolic features of the M2 phenotype, and M2 macrophages are involved in the resolution of inflammation and therefore do not need to produce energy in a rapid manner but need continuously; therefore, M2 macrophages whose metabolism is mainly enhanced fatty acid oxidation (FAO) as well as oxidative phosphorylation (OXPHOS) [51, 87] have an intact TCA cycle. IL-6/JAK/STAT3 has an important role in the M2 phenotypic polarization of macrophages, and M2 polarization can be mediated by IL-6/JAK2/STAT3 pathway activation [88, 89]. Wnt β-catenin Signaling has an important role in the regulation of macrophage polarization, and it was found that activating Wnt2b/β-catenin/c-Myc signaling promotes the polarization of TAM to M2-like macrophages. In addition, activation of the Wnt/β-catenin signaling pathway inhibits macrophage M1 polarization and promotes the polarization of RAW264.7 cells to the M2 phenotype [90, 91]. Many studies have shown that the TGF-β signaling pathway has an immunosuppressive effect on macrophage polarization. Hh decreases the flux through the UDP-GlcNAc biosynthetic pathway. Thus, reduced O-GlcNAc modification of STAT6 attenuated the immunosuppressive program of M2 macrophages [92]. Since STAT6 plays a dual role in regulating the M2 phenotype and fatty acid oxidation, this ultimately leads M2 macrophages to shift their metabolism and bioenergetics from fatty acid oxidation to glycolysis, metabolically altering the M2 phenotype to resemble M1.

Significant responses or pathways by which glycopeptides dCP1 regulate the polarization of M2-like TAM to M1 phenotype were found to be associated with PIK3 and PI3K-AKT pathways by KEGG analysis and Hub gene analysis. PI3K is a key molecule in the signal transduction pathway initiated by extracellular signal binding to cell surface receptors with serine/threonine kinase activity and phosphatidylinositol kinase activity. In macrophages, the PI3K/Akt pathway transduces signals from a variety of receptors, including insulin receptors (IRs), cytokine and adipokine receptors, and receptors necessary for the induction of activation of innate immunity. Thus, activation of the PI3K/Akt pathway coordinates the response to different metabolic and inflammatory signals in macrophages [93], and the PI3K/Akt pathway and its downstream targets have become central regulators of the macrophage activation phenotype in different signaling cascades. In addition to this, the PI3K-AKT pathway is also an important regulatory pathway for lipid metabolism, such as the synthesis and secretion of triglycerides. For instance, tetramethylpyrazine can inhibit lipid accumulation in macrophages by downregulating scavenger receptors and upregulating ATP-binding cassette transport proteins via PI3K/Akt and p38/MAPK signaling [94].

Metabolites are key biological communication channels of the organism. Physiological conditions are defined by complex metabolic pathways and metabolites, and the metabolic adaptation of macrophages is closely related to their primary function. Lipids are key metabolites in the process of macrophage polarization, and lipid metabolism has important influences on the polarization of macrophages. Analysis of lipid metabolite content in M2-like TAM before and after dCP1 intervention revealed M2-like TAM after treatment with glycopeptides, the lipid metabolites like SM, Cer [69, 95–98], TG, DG, MG [99–101], LPS [102], etc., which were at higher content levels in M1 phenotype than in M2 phenotype or pro-inflammatory lipid metabolites were increased; and lipid metabolites like PS [103, 104], Pet, PI, PC [68–70], etc., which were at higher content levels in M2 phenotype than in M1 phenotype were decreased. The M2-like TAM polarized toward the M1 phenotype after treatment with the glycopeptide dCP1 when compared with the lipid metabolic profile of M1 phenotype macrophages. The interconversion between PE and PC, and PC and DG is core reactions of key differential metabolic. Phosphatidylcholine can be synthesized from phosphatidylethanolamine through the Kennedy pathway, which is also known as the CDP-choline pathway. It involves a series of enzymatic reactions that transfer three methyl groups to phosphatidylethanolamine, leading to the formation of phosphatidylcholine [105]. Phosphatidylcholine (PC) can be converted to diglyceride (DG) through the activity of the enzyme phospholipase C (PLC), like PC-PLC [106]. PLC cleaves the phosphodiester bond of PC to produce DG [107]. PC-PLC can be involved in macrophage activation reactions [106], and the second messengers DG and Ca^2+^ initiate membrane translocation and activation of PKC. PKC has an important role for macrophage activation and immune response.

The glycopeptides in dCP1 have a low molecular weight (1.2–5 K), which makes them possible to be directly absorbed, then identified, and bound by receptors on TAM that can recognize glycoproteins. The receptors expressed by macrophages that can recognize and bind to glycoproteins or glycopeptides are mainly Toll-like receptor 2, NOD1/2, and part of the C-type lectin superfamily. Molecular docking of glycopeptides to these receptors revealed that glycopeptides had the highest binding score to TLR2 receptors. It was shown that multiple TLR stimulators were able to activate the PI3K-Akt signaling pathway along with the activation of the TLR pathway. It has been reported that the cascade signaling response consisting of TLR2-Rac1-PI3K-Akt can promote the trans-activation of p65 in a manner independent of IκB degradation and promote the transcription of p65-regulated inflammatory factors thereby promoting the inflammatory response; in 293-TLR2 and THP1 cells, *S. aureus* induced the activation of TLR2 and Rac1 and the TLR2- Rac1-P85 complex formation, which was recruited to PI3K activation of the TLR2 complex, further promoting Akt activation [108]. The molecular dynamics simulations of the glycopeptides with the highest and lowest docking scores were consistent with the molecular docking results. Regrettably, we did not synthesize the highest scoring glycopeptides to verify whether it binds to TLR2 to play a role in regulating the M2-like TAM phenotype, but only verified the results obtained by molecular docking through molecular dynamics simulations. The reason is that due to the limitations of current technology, there are still many challenges in synthesizing glycopeptides that fully conform to the specified structure [109]. The structure of glycopeptides is more complex than that of ordinary polypeptides, because glycans have more abundant and diverse chemical properties than amino acid side chains, and there are specific stereo-conformational effects between glycans and amino acids. These specific properties make it extremely difficult to synthesize glycopeptides that fully conform to the specified structure. Although the linear structure and sequence in glycopeptides synthesis can be controlled, unavoidable errors may occur during synthesis, inactivation, separation, and side reactions may occur during steps such as substitution reactions, deprotection reactions, or polymerization reactions, or its spatial structure changes due to conformational switching or corner effects, so the glycopeptides obtained after synthesis cannot be guaranteed to be completely consistent with its original spatial structure, which may cause the synthesized glycopeptides to behave differently from the original glycopeptides activity and efficacy [110, 111]. When the technology develops more maturely, we will get glycopeptides with exactly the same structure and conduct experimental verification.

In the tumor microenvironment, most TAM are M2 phenotype macrophages that promote tumor growth and metastasis, and only a few TAM are M1 phenotypes that inhibit tumor cell growth. M2-like TAM is the traitor of the immune system of tumor patients and is one of the accomplices that promote the growth and metastasis of tumor cells. If these "accomplices" and "traitors" can be transformed into "defenders" of the body's health by regulating immunity, the tumor treatment and prognosis are a positive sign. Overall, our study demonstrated that glycopeptide dCP1 can polarize M2-like TAMs toward the M1 phenotype at the level of transcription and lipid metabolism and may regulate the PI3K-AKT signaling pathway through the binding of glycopeptides to TLR2.

### Supplementary Information

Below is the link to the electronic supplementary material.

Supplementary file1 (DOCX 9088 kb)
