# Supplementary material for: Codonopsis pilosula-derived glycopeptide dCP1 promotes the polarization of tumor-associated macrophage from M2-like to M1 phenotype
Source: Cancer Immunol Immunother. 2024 May 14;73(7):128. doi: 10.1007/s00262-024-03694-6 (PMC11093951; doi:10.1007/s00262-024-03694-6)
Supplement: Supplementary file 1 — Supplementary file1 (DOCX 9088 kb) [file 262_2024_3694_MOESM1_ESM.docx]

**Supplementary Material**

**Supplementary-Tables**

Table S1 Primer sequences

| Gene | Forward primers (5′-3′) | Reverse primers (3′-5′) |
| --- | --- | --- |
| *β-actin* | GGCTGTATTCCCCTCCATCG | CCAGTTGGTAACAATGCCATGT |
| *IL-1β* | GCAACTGTTCCTGAACTCAACT | ATCTTTTGGGGTCCGTCAACT |
| *IL-6* | ACACACTGGTTCTGAGGGAC | TACCACAAGGTTGGCAGGTG |
| *iNOS* | GTTCTCAGCCCAACAATACAAGA | GTGGACGGGTCGATGTCAC |
| *TNF-α* | CCCTCACACTCAGATCATCTTCT | GCTACGACGTGGGCTACAG |
| *Mrc1* | AGGGACCTGGATGGATGACA | TGTACCGCACCCTCCATCTA |
| *Arg1* | CTCCAAGC CAAAGTCCTTAGAG | GGAGCTGTCATTAGGGACATCA |
| *Fizz1* | CCAATCCAGCTAACTATCCCTCC | ACCCAGTAGCAGTCATCCCA |

Table S2 Glycopeptides Information

| Peptide | Type | Glycans | Modification Type(s) | m/z | Observed (M+H) | Calc.mass (M+H) | Score | Intensity |
| --- | --- | --- | --- | --- | --- | --- | --- | --- |
| ITN*K | N-Glyco | HexNAc(4)Hex(3)Fuc(1)Pent(1) | N[+1577] | 1026.4393 | 2051.8712 | 2051.8636 | 170.36 | 4775200 |
| CNSLLKVLNN*VTDQGELR | N-Glyco | HexNAc(2)Hex(2) | N[+730] | 935.1324 | 2803.3826 | 2803.3401 | 46.8 | 2301500 |
| VN*CTFISHHAIGK | N-Glyco | HexNAc(4)Hex(3)Fuc(1) | N[+1445] | 976.7605 | 2928.2668 | 2928.2813 | 55.71 | 66963000 |
| LYDVGFSHLPAN*QTK | N-Glyco | HexNAc(2)Hex(2) | N[+730] | 807.386 | 2420.1436 | 2420.1239 | 100.15 | 143060000 |
| VVVVN*R | N-Glyco | HexNAc(3)Hex(5)Fuc(1) | N[+1566] | 750.9998 | 2250.985 | 2250.9957 | 61.25 | 225830000 |
| ELGYTN*KSPR | N-Glyco | HexNAc(4)Hex(5)Fuc(1) | N[+1769] | 489.7124 | 2933.2381 | 2933.2403 | 20.38 | 476800000 |
| QVQLN*LTSR | N-Glyco | HexNAc(2)Hex(1) | N[+568] | 813.9114 | 1626.8156 | 1626.8069 | 88.18 | 155500000 |
| EILAN*SSLR | N-Glyco | HexNAc(2) | N[+406] | 470.2438 | 1408.7169 | 1408.7166 | 142.27 | 64819000 |
| TLN*K | N-Glyco | HexNAc(4)Hex(3)Fuc(1)Pent(1) | N[+1577] | 1026.4393 | 2051.8712 | 2051.8636 | 170.36 | 4775200 |
| KYSCEVYS*VEQALK | O-Glyco | HexNAc(3)Hex(2)Fuc(1) | S[+1079] | 464.7106 | 2783.2273 | 2783.2326 | 12.77 | 2670600 |
| EFLES*IQR | O-Glyco | HexNAc(3)Hex(2)Fuc(3) | S[+1372] | 798.3582 | 2393.06 | 2393.0488 | 56.48 | 2129300000 |
| QHYRS*R | O-Glyco | HexNAc(1)Hex(1) | S[+365] | 404.5256 | 1211.5623 | 1211.5651 | 251.13 | 176020000 |
| S*QDLNK | O-Glyco | HexNAc(5)Hex(5)Fuc(3) | S[+2264] | 990.0568 | 2968.1559 | 2968.1921 | 0.22 | 233850000 |
| SVPS*HPHQFR | O-Glyco | HexNAc(3) | S[+609] | 600.9514 | 1800.8397 | 1800.8399 | 39.77 | 1974300 |
| VIT*SIEQLPEWNFDGSSTNQAPGYDSDIYLK | O-Glyco | HexNAc(1)Hex(2)Fuc(1) | T[+673] | 832.9849 | 4160.8956 | 4160.8969 | 93.31 | 109980000 |
| NIAMPLNTIIEINKKIVIT*SLLSIIEK | O-Glyco | HexNAc(4)Hex(1)Fuc(1) | T[+1120] | 691.3782 | 4143.2328 | 4142.2278 | 5.13 | 766760000 |
| FCGVIT*K | O-Glyco | HexNAc(3)Fuc(1) | T[+755] | 527.2509 | 1579.738 | 1579.7295 | 122.52 | 163810000 |
| HFPDTAS*EYENIDSR | O-Glyco | HexNAc(6)Hex(5)Fuc(3) | S[+2467] | 472.9633 | 4248.6117 | 4247.6914 | 49.2 | 104460000 |
| S*VVAAEDAK | O-Glyco | HexNAc(3)Hex(3) | S[+1095] | 662.2939 | 1984.867 | 1984.8591 | 126.81 | 451860000 |
| DMPS*LK | O-Glyco | HexNAc(2)Hex(3) | S[+892] | 528.2168 | 1582.6358 | 1582.6663 | 52.48 | 37374000 |
| AVGRGLVS*SCICVGR | O-Glyco | HexNAc(3) | S[+609] | 550.7723 | 2200.0674 | 2200.0584 | 239.75 | 27326000 |
| EDS*CECK | O-Glyco | HexNAc(3)Fuc(1) | S[+755] | 561.5433 | 1682.6153 | 1682.6143 | 90.13 | 360330000 |
| DLEDSPVRIT*AYIEDVDALAQAGADIIAIDGTDR | O-Glyco | HexNAc(5)Hex(5)Fuc(3) | T[+2264] | 534.0558 | 5864.5409 | 5865.6215 | 11.58 | 96193000 |
| T*GER | O-Glyco | HexNAc(2)Hex(2)Fuc(2) | T[+1022] | 495.5402 | 1484.6059 | 1484.6109 | 98.94 | 124350000 |
| AAGISQAQART*IGIAVDVR | O-Glyco | HexNAc(5)Hex(5)Fuc(3) | T[+2264] | 694.321 | 4160.8897 | 4160.8961 | 90.06 | 492330000 |
| DYAIPT*AQLLLVWRDFDSR | O-Glyco | HexNAc(4)Hex(4)Fuc(3) | T[+1899] | 696.9849 | 4176.873 | 4177.8844 | 0.79 | 581300000 |
| DLQDAS*VAIENR | O-Glyco | HexNAc(3)Hex(3) | S[+1095] | 607.2722 | 2426.0669 | 2426.0563 | 14.46 | 1355600 |
| RPELDAT*LVAK | O-Glyco | HexNAc(2)Hex(2) | T[+730] | 486.5037 | 1942.9928 | 1942.9591 | 137.04 | 21239000 |
| IQESRS*QLSNK | O-Glyco | HexNAc(4)Hex(3)Fuc(2) | S[+1591] | 960.763 | 2880.2745 | 2880.2726 | 139.82 | 945880000 |
| FYSQMISESFSIIVEIPFS*LR | O-Glyco | HexNAc(6)Hex(5)Fuc(3) | S[+2467] | 827.3755 | 4959.2169 | 4960.1875 | 5.51 | 379160000 |
| WPQYLQT*NGLAEPQKSNR | O-Glyco | HexNAc(3)Hex(1) | T[+771] | 967.8096 | 2901.4142 | 2901.3636 | 114.4 | 300850000 |
| DLLQPSS*PLDIPGR | O-Glyco | HexNAc(4)Hex(1)Fuc(1) | S[+1120] | 657.8159 | 2628.2419 | 2628.2397 | 149.3 | 74169000 |
| TLIPVEKDVDCLT*TGGAAK | O-Glyco | HexNAc(3) | T[+609] | 866.4335 | 2597.2859 | 2597.275 | 70.26 | 69985000 |
| GPFS*DK | O-Glyco | HexNAc(2)Hex(1)Fuc(2) | S[+860] | 1510.6401 | 1510.6401 | 1510.6418 | 47.68 | 177800000 |
| YYLITLGT*GIVPLFLFHTIR | O-Glyco | HexNAc(2)Hex(2)Fuc(1) | T[+876] | 536.4535 | 3213.6847 | 3213.6592 | 6.32 | 132690000 |
| DPDKAFLPT*LLAR | O-Glyco | HexNAc(3)Hex(2)Fuc(2) | T[+1225] | 894.7613 | 2682.2693 | 2682.2754 | 0.77 | 80250000 |
| CMGPT*AQRR | O-Glyco | HexNAc(3) | T[+609] | 562.588 | 1685.7495 | 1685.7469 | 135.22 | 2236500000 |
| ERIFISVLGVGGS*ILGCFAFSK | O-Glyco | HexNAc(3)Hex(3)Fuc(2)NeuAc(2) | S[+1970] | 481.5653 | 4326.0294 | 4326.9718 | 49.69 | 63129000 |
| DLVPESQAYMDLLT*FER | O-Glyco | HexNAc(5)Hex(3) | T[+1502] | 392.9552 | 3528.5387 | 3528.5344 | 14.99 | 234150000 |
| IVALT*NAK | O-Glyco | HexNAc(3)Fuc(1) | T[+755] | 528.9436 | 1584.8163 | 1584.8102 | 173.94 | 210340000 |
| NLGS*VAGPR | O-Glyco | HexNAc(1)Hex(1) | S[+365] | 618.3065 | 1235.6058 | 1235.6114 | 259.09 | 85919000 |
| GT*QDLFLAR | O-Glyco | HexNAc(2) | T[+406] | 476.2416 | 1426.7102 | 1426.706 | 172.59 | 1506000 |
| EAIQLAYYEGLTYVQVSERLSANLAT*IK | O-Glyco | HexNAc(4)Hex(2) | T[+1136] | 476.573 | 4281.0989 | 4280.0854 | 22.2 | 476480000 |
| CGDS*MDDTFFSESQR | O-Glyco | HexNAc(6)Hex(5)Fuc(3) | S[+2467] | 425.6677 | 4247.6113 | 4248.5882 | 35 | 25353000 |
| LDVT*LEEWDLSGLPWWFLGNLR | O-Glyco | HexNAc(3)Hex(2)Fuc(3) | T[+1372] | 672.6638 | 4030.9467 | 4030.873 | 69.77 | 129630000 |
| LEFYSVDDLS*GIVSR | O-Glyco | HexNAc(2)Hex(2) | S[+730] | 405.8601 | 2430.1242 | 2430.1181 | 20.35 | 32638000 |
| FHQGT*LLVLAIPEHQIATQEQPAFDR | O-Glyco | HexNAc(5)Hex(5) | T[+1826] | 798.5429 | 4786.2212 | 4785.2034 | 7.8 | 102810000 |
| NS*EPVDLR | O-Glyco | HexNAc(3)Hex(3)Fuc(2) | S[+1388] | 773.0003 | 2316.9863 | 2316.9811 | 26.65 | 275800000 |
| YLDNLS*EEDVLK | O-Glyco | HexNAc(2)Hex(2)Fuc(2) | S[+1022] | 820.7 | 2460.0854 | 2460.091 | 43.88 | 150800000 |
| FISEVENTDPTQERVNVIGGHSGITIIPLIS*QTNHK | O-Glyco | HexNAc(4)Hex(1) | S[+974] | 1640.1382 | 4918.4 | 4918.4215 | 45.39 | 2447300000 |
| GYGASAQAALVT*R | O-Glyco | HexNAc(2) | T[+406] | 835.9142 | 1670.8211 | 1670.8232 | 212.52 | 231530000 |
| SES*IVK | O-Glyco | HexNAc(4)Hex(1)Fuc(1) | S[+1120] | 891.9008 | 1782.7942 | 1782.8002 | 85.29 | 1793000 |
| LTES*FNLMLR | O-Glyco | HexNAc(3)Fuc(1) | S[+755] | 660.3208 | 1978.9478 | 1978.9413 | 141.26 | 1590600000 |
| LAVITFHS*LEDRLIK | O-Glyco | HexNAc(3)Fuc(1) | S[+755] | 628.3333 | 2510.3115 | 2510.3124 | 88.02 | 433610000 |
| EKPT*PSDFPR | O-Glyco | HexNAc(5)Hex(5)Fuc(3) | T[+2264] | 573.7442 | 3437.4289 | 3437.4246 | 8.19 | 178570000 |
| ALDDLAQLAS*QPK | O-Glyco | HexNAc(3) | S[+609] | 660.3394 | 1979.0036 | 1978.9703 | 115.29 | 2778100000 |
| LSGPGWDVS*TR | O-Glyco | HexNAc(1)Hex(1) | S[+365] | 1539.724 | 1539.724 | 1539.7173 | 57.85 | 231120000 |
| ESVQYADRCFWFTS*LVAR | O-Glyco | HexNAc(4)Hex(4)Fuc(3) | S[+1899] | 460.0917 | 4132.7675 | 4133.7677 | 32.28 | 87779000 |
| QLARDGVT*IFFTQVFR | O-Glyco | HexNAc(3)Fuc(1) | T[+755] | 443.0593 | 2653.3193 | 2653.3243 | 10.15 | 9276100 |
| HLIPVVS*SLFLTFAR | O-Glyco | HexNAc(4)Hex(1)Fuc(1) | S[+1120] | 470.9098 | 2820.4223 | 2820.4176 | 8.57 | 42735000 |
| ADLFS*VRLAK | O-Glyco | HexNAc(2)Hex(3) | S[+892] | 671.3299 | 2011.975 | 2011.9693 | 127.71 | 133460000 |
| EIFENAMDAIIIWS*NDGR | O-Glyco | HexNAc(6)Hex(5)Fuc(3) | S[+2467] | 456.9969 | 4560.9038 | 4560.9101 | 46.13 | 145560000 |
| ELGFIS*KAPR | O-Glyco | HexNAc(3)Hex(1) | S[+771] | 630.3134 | 1888.9258 | 1888.9274 | 206.54 | 254380000 |
| QPWWEQNNIIS*ALYWYLTDTFEPSQR | O-Glyco | HexNAc(3)Hex(2) | S[+933] | 844.3899 | 4217.9205 | 4218.9078 | 61.24 | 1286200 |
| RPCGGFQDS*LSPR | O-Glyco | HexNAc(6)Hex(5)Fuc(3) | S[+2467] | 658.1051 | 3943.594 | 3943.6153 | 33.24 | 1052700000 |
| CGYACS*DHASADR | O-Glyco | HexNAc(6)Hex(5)Fuc(3) | S[+2467] | 492.9379 | 3936.4527 | 3936.4673 | 39.51 | 377260000 |
| SFMCT*VCGR | O-Glyco | HexNAc(2)Hex(2)Fuc(1) | T[+876] | 399.5638 | 1993.7899 | 1993.781 | 24.43 | 9370600000 |
| NYT*VPIK | O-Glyco | HexNAc(3)Hex(1)Fuc(2) | T[+1063] | 633.3002 | 1897.886 | 1897.8787 | 158.94 | 2397200000 |
| T*LGGLLRFK | O-Glyco | HexNAc(4)Hex(1) | T[+974] | 660.3363 | 1978.9944 | 1978.9954 | 137.06 | 1679100 |
| HPEYAVS*VLLR | O-Glyco | HexNAc(4)Hex(1)Fuc(1) | S[+1120] | 401.5244 | 2404.1102 | 2404.1389 | 88.08 | 14289000 |
| DEYVVTDYSVELPDS*PPGSR | O-Glyco | HexNAc(4)Hex(4)Fuc(3) | S[+1899] | 688.1381 | 4123.7922 | 4123.727 | 38.81 | 124510000 |
| AVT*GSLK | O-Glyco | HexNAc(3)Hex(2)Fuc(2) | T[+1225] | 634.2926 | 1900.8632 | 1900.8631 | 104.07 | 232250000 |
| LMPEDAILTT*DIGNVASTANSYFK | O-Glyco | HexNAc(2)Hex(2) | T[+730] | 1101.1828 | 3301.5338 | 3301.5291 | 26.55 | 173900000 |
| EYGLDAFET*R | O-Glyco | HexNAc(6)Hex(5)Fuc(3) | T[+2467] | 612.0832 | 3667.4629 | 3667.4672 | 41.7 | 2001700000 |
| LAGIEVSLTEIRRILNHLGFTVVGQAPVVKVAVPS*WR | O-Glyco | HexNAc(3)Hex(1)Fuc(2) | S[+1063] | 1696.6031 | 5087.7949 | 5088.7242 | 22.35 | 1980300000 |
| SQIHIS*QPQFLK | O-Glyco | HexNAc(2)Hex(1)Fuc(1) | S[+714] | 714.0238 | 2140.0568 | 2140.0544 | 11.17 | 157100000 |
| DALGVT*GDQVWTFAK | O-Glyco | HexNAc(2) | T[+406] | 671.9934 | 2013.9656 | 2013.9651 | 127.15 | 106250000 |
| GEAFAT*AVRAVIR | O-Glyco | HexNAc(3)Hex(2) | T[+933] | 765.3777 | 2294.1184 | 2294.1133 | 50.08 | 147260000 |
| ADVVKQGAKVAILS*FGTLLENAQLAADELNATLVNMR | O-Glyco | HexNAc(1)Hex(2)Fuc(1) | S[+673] | 1520.1155 | 4558.3321 | 4557.3379 | 13.25 | 150850000 |
| PRDTTTYDS*R | O-Glyco | HexNAc(3)Fuc(1) | S[+755] | 656.2909 | 1966.858 | 1966.8611 | 128.68 | 130850000 |
| PEVIET*YWYLWR | O-Glyco | HexNAc(2)Hex(3)Fuc(1) | T[+1038] | 674.0568 | 2693.2053 | 2693.2015 | 58.33 | 73224000 |
| CDS*R | O-Glyco | HexNAc(3)Hex(3)Fuc(3) | S[+1534] | 690.9301 | 2070.7759 | 2070.7789 | 127.6 | 72726000 |
| MMEMETQNQINSNQLRRAEQEVNS*LQEK | O-Glyco | HexNAc(3)Hex(3) | S[+1095] | 1491.9965 | 4473.9748 | 4473.9801 | 15.42 | 1412500000 |
| GEQSCT*CR | O-Glyco | HexNAc(1) | T[+203] | 600.7379 | 1200.4684 | 1200.462 | 157.31 | 555790000 |
| EAS*IPLGILVVR | O-Glyco | HexNAc(3)Hex(3)Fuc(1) | S[+1241] | 418.8779 | 2508.2311 | 2508.2325 | 23.36 | 38176000 |
| T*IPYLTDFK | O-Glyco | HexNAc(3)Hex(3) | T[+1095] | 731.6745 | 2193.009 | 2192.9843 | 9.47 | 393090000 |
| NDT*VELENQQR | O-Glyco | HexNAc(4)Hex(1)Fuc(1) | T[+1120] | 822.6998 | 2466.0848 | 2466.0625 | 117.49 | 702790 |
| VQGLLPS*MVK | O-Glyco | HexNAc(1)Hex(1)Fuc(1) | S[+511] | 528.2764 | 1582.8145 | 1582.8132 | 172.55 | 660410000 |
| GLLPS*LFYK | O-Glyco | HexNAc(1)Hex(1)Fuc(1)NeuGc(1) | S[+818] | 619.2996 | 1855.8842 | 1855.8834 | 169.53 | 118340000 |
| AFSSGLTDVIYLLEDPLAGIHPKDAPS*LLSVIK | O-Glyco | HexNAc(3)Hex(3) | S[+1095] | 763.224 | 4574.3078 | 4575.3002 | 23.1 | 402110000 |
| CAPGT*CQNLDGSYR | O-Glyco | HexNAc(1) | T[+203] | 901.3824 | 1801.7576 | 1801.748 | 142.47 | 8851000 |
| DSCCT*K | O-Glyco | HexNAc(3) | T[+609] | 690.2643 | 1379.5214 | 1379.5189 | 61.63 | 846260 |
| PNSVET*AETK | O-Glyco | HexNAc(3)Hex(3)Fuc(1) | T[+1241] | 772.9976 | 2316.9782 | 2316.9811 | 54.11 | 395470000 |

Table S3 M1/M2 marker genes

| M1 marker gene | M2 marker gene |
| --- | --- |
| *Aif1、Cd80、Cd86、Socs3、Ccr7、Cybb、Fcgr1、Fcgr3、Gapdh、*  *Ikbkb、Il12b、Il1b、Il6、Nos2、*  *Ccl2、Ccl8、Ccl9、Sphk1、Ccl3、Ccl4、Ccl5、Stat1、Tlr4、Tnf、*  *Tlr2、Slamf1、Il23a* | *Arg1、Chil3、Ccr2、Col14a1、Fcgr2b、Cxcl10、Il10、Il1r2、Il4ra、Itgam、Tlr8、Marco、Mrc1、Pparg、Ccl22、Ccl1、Ccl11、Ccl17、Stat6、Tgfb1、Tgm2、Vegfa、Vegfb、Cd200r1、Il27ra、Ccl24、Retnla、Cxcl16、Trem2、Cd163、Chil4、Il1rn、Ptgs2、*  *Msr1、Chil4* |

Table S4 Hub genes screen and score

| Rank | Gene | Score |
| --- | --- | --- |
| 1 | *Hras* | 114 |
| 1 | *Src* | 114 |
| 3 | *Mtor* | 112 |
| 4 | *Trp53* | 110 |
| 5 | *Gapdh* | 102 |
| 6 | *Pik3ca* | 97 |
| 7 | *Ctnnb1* | 96 |
| 8 | *Stat3* | 95 |
| 9 | *Pten* | 93 |
| 10 | *Pik3r1* | 91 |

Table S5 Reactions between Lipid metabolites in the optimal subnetwork

| Identifier | Reaction | Enzyme class or enzymes |
| --- | --- | --- |
| RHEA:36231 | 1-O-alkyl-2-acyl-sn-glycero-3-phosphocholine + H_2_O = 1-O-alkyl-sn-glycero-3-phosphocholine + a fatty acid + H^+^ | EC 3.1.1.4 phospholipase A2 |
| RHEA:44068 | 1-O-(1Z-alkenyl)-2-acyl-sn-glycero-3-phosphocholine + H_2_O => 1-O-(1Z-alke nyl)-sn-glycero-3-phosphocholine + a fatty acid + H^+^ | Calcium-independent phospholipase A2-gamma |
| RHEA:40579 | 1-O-(1Z)-hexadecenyl-2 (5Z,8Z,11Z,14Z)-eicosatetraenoyl-sn-glycero-3-phosphocholine + H_2_O = (5Z,8Z,11Z,14Z)-eicosatetraenoate + 1-(1Z-hexadecenyl)-sn-glycero-3-phosphocholine + H^+^ | Calcium-independent phospholipase A2-gamma |
| RHEA:41067 | 1-O-hexadecyl-2-(5Z,8Z,11Z,14Z)-eicosatetraenoyl-sn-glycero-3-phosphocholine + H_2_O => (5Z,8Z,11Z,14Z)-eicosatetraenoate + 1-O-hexadecyl-sn-glycero-3-phos | Cytosolic phospholipase A2 zeta、  85/88 kDa calcium-independent phospholipase A2、  Cytosolic phospholipase A2、  Cytosolic phospholipase A2 gamma |
| RHEA:41271 | 1-(5Z,8Z,11Z,14Z-eicosatetraenoyl)-2-O-hexadecyl-sn-glycero-3-phosphocholine + H_2_O = (5Z,8Z,11Z,14Z)-eicosatetraenoate + 2-O-hexadecyl-sn-glycero-3-phosphocholine + H^+^ | Cytosolic phospholipase A2 |
| RHEA:23992 | 1-O-alkyl-sn-glycero-3-phosphocholine + an acyl-CoA = 1-O-alkyl-2-acyl-sn-glycero-3-phosphocholine + CoA | EC:2.3.1.63  1-alkylglycerophosphocholine O-acyltransferase |
| RHEA:41692 | 1-O-hexadecyl-2-butanoyl-sn-glycero-3-phosphocholine + H_2_O = 1-O-hexadecyl-sn-glycero-3-phosphocholine + butanoate + H^+^ | Platelet-activating factor acetylhydrolase 2, cytoplasmic |
| RHEA:11048 | 1-organyl-2-lyso-sn-glycero-3-phospholipid + a 1-O-alkyl-2-acetyl-sn-glycero-3-phosphocholine = 1-O-alkyl-sn-glycero-3-phosphocholine + 1-organyl-2-acetyl-sn-glycero-3-phospholipid | EC:2.3.1.149  platelet-activating factor acetyltransferase |
| RHEA:17777 | a1-O-alkyl-2-acetyl-sn-glycero-3-phosphocholine + H_2_O = 1-O-alkyl-sn-glycero-3-phosphocholine + acetate + H^+^ | EC:3.1.1.47 1-alkyl-2-acetylglycerophosphocholine esterase |
| RHEA:10344 | 1-O-(1Z-alkenyl)-sn-glycero-3-phosphocholine + an acyl-CoA = 1-O-(1Z-alkenyl)-2-acyl-sn-glycero-3-phosphocholine + CoA | EC:2.3.1.25 plasmalogen synthase |
| RHEA:40915 | 1-O-hexadecyl-2-(9Z)-octadecenoyl-sn-glycero-3-phosphocholine + H_2_O = (9Z)-octadecenoate + 1-O-hexadecyl-sn-glycero-3-phosphocholine + H^+^ | Phospholipase B1, membrane-associated |
| RHEA:41688 | 1-O-hexadecyl-2-propanoyl-sn-glycero-3-phosphocholine + H_2_O = 1-O-hexadecyl-sn-glycero-3-phosphocholine + H^+^ + propanoate | Platelet-activating factor acetylhydrolase 2, cytoplasmic |
| RHEA:41696 | 1-O-hexadecyl-2-succinyl-sn-glycero-3-phosphocholine + H_2_O = 1-O-hexadecyl-sn-glycero-3-phosphocholine + H^+^ + succinate | Platelet-activating factor acetylhydrolase 2, cytoplasmic |
| RHEA:41700 | 1-O-hexadecyl-2-glutaryl-sn-glycero-3-phosphocholine + H_2_O = 1-O-hexadecyl-sn-glycero-3-phosphocholine + glutarate + H^+^ | Platelet-activating factor acetylhydrolase 2, cytoplasmic |
| RHEA:54552 | 1-O-hexadecyl-2-nonadioyl-sn-glycero-3-phosphocholine + H_2_O = 1-O-hexadecyl-sn-glycero-3-phosphocholine + H^+^ + nonanedioate | Phospholipase ABHD3 |

Table S6 Key differential lipid metabolites

| Lipid Metabolites | Molecular formula | VIP value | | Fold change (dCP1 vs control) | *P* value |
| --- | --- | --- | --- | --- | --- |
| PC (18:1/18:2) | C_44_H_83_O_8_N_1_P_1_ | 5.69488 | 0.56332 | | 0.001 |
| SM (d16:0/18:1) | C_39_H_80_O_6_N_2_P_1_ | 4.39568 | 1.26878 | | 0.001 |
| PC (16:0/16:0) | C_40_H_81_O_8_N_1_P_1_ | 4.33112 | 1.69629 | | ＜0.001 |
| DG (18:1/18:2) | C_39_H_74_O_5_N_1_ | 4.29241 | 1.23306 | | 0.008 |
| DG (18:1/18:1) | C_39_H_76_O_5_N_1_ | 4.05076 | 1.34847 | | 0.001 |
| PC (18:1/18:1) | C_44_H_85_O_8_N_1_P_1_ | 3.94027 | 0.73613 | | 0.004 |
| PC (18:0/16:0) | C_42_H_85_O_8_N_1_P_1_ | 3.30281 | 1.44105 | | ＜0.001 |
| PC (33:2) | C_41_H_79_O_8_N_1_P_1_ | 2.75166 | 0.32148 | | ＜0.001 |
| Cer (d18:1/24:1) | C_42_H_82_O_3_N_1_ | 2.45465 | 1.60702 | | ＜0.001 |
| Cer (d18:1/16:0) | C_34_H_68_O_3_N_1_ | 2.37916 | 2.28896 | | ＜0.001 |
| Cer(d34:0) | C_34_H_70_O_3_N_1_ | 2.17103 | 1.74580 | | 0.001 |
| DG (16:0/18:1) | C_37_H_74_O_5_N_1_ | 2.14323 | 1.29592 | | 0.001 |
| PE (18:0/18:1) | C_41_H_81_O_8_N_1_P_1_ | 2.13039 | 0.42129 | | ＜0.001 |
| LPC (16:0) | C_24_H_51_O_7_N_1_P_1_ | 2.11139 | 5.60104 | | 0.001 |
| PE (18:0p/18:2) | C_41_H_79_O_7_N_1_P_1_ | 2.05343 | 0.34763 | | ＜0.001 |
| LPC(20:0) | C_28_H_59_O_7_N_1_P_1_ | 2.05206 | 31.44673 | | 0.001 |
| DG (18:0/18:1) | C_39_H_78_O_5_N_1_ | 2.04461 | 1.56535 | | 0.002 |
| DG (18:1/20:2) | C_41_H_78_O_5_N_1_ | 1.95953 | 1.31165 | | 0.004 |
| PC (18:2/18:2) | C_44_H_81_O_8_N_1_P_1_ | 1.90744 | 0.78750 | | 0.002 |
| PC(33:3) | C_41_H_77_O_8_N_1_P_1_ | 1.73032 | 0.18212 | | ＜0.001 |
| PE(34:0) | C_39_H_78_O_8_N_1_P_1_Na_1_ | 1.69222 | 0.21318 | | ＜0.001 |
| PE (18:1/18:2) | C_41_H_77_O_8_N_1_P_1_ | 1.64955 | 0.18393 | | 0.001 |
| PS (18:0/18:1) | C_42_H_81_O_10_N_1_P_1_ | 1.60266 | 0.25647 | | 0.008 |
| DG (18:0/20:2) | C_41_H_80_O_5_N_1_ | 1.52893 | 1.47697 | | 0.002 |
| PC (20:0/18:2) | C_46_H_89_O_8_N_1_P_1_ | 1.51012 | 1.20760 | | 0.001 |
| PC (18:1/18:2) | C_45_H_83_O_10_N_1_P_1_ | 1.50416 | 0.10932 | | 0.001 |
| PS (18:0/18:2) | C_42_H_79_O_10_N_1_P_1_ | 1.50097 | 0.15130 | | 0.001 |
| PE (16:0p/18:2) | C_39_H_75_O_7_N_1_P_1_ | 1.47915 | 0.24575 | | ＜0.001 |
| LPC (16:0p) | C_24_H_51_O_6_N_1_P_1_ | 1.47485 | 17.47697 | | ＜0.001 |
| PC (18:0/20:3) | C_46_H_87_O_8_N_1_P_1_ | 1.47207 | 0.82410 | | 0.001 |
| PE (18:0/22:4) | C_45_H_83_O_8_N_1_P_1_ | 1.43829 | 0.34592 | | 0.001 |
| PC (30:0) | C_38_H_77_O_8_N_1_P_1_ | 1.37124 | 1.41442 | | ＜0.001 |
| PE (18:0p/22:4) | C_45_H_83_O_7_N_1_P_1_ | 1.36434 | 0.39479 | | ＜0.001 |
| PE (16:0p/20:1) | C_41_H_81_O_7_N_1_P_1_ | 1.36041 | 0.41305 | | ＜0.001 |
| PE (18:0/20:3) | C_43_H_81_O_8_N_1_P_1_ | 1.36008 | 0.20068 | | ＜0.001 |
| LPC (18:0) | C_26_H_55_O_7_N_1_P_1_ | 1.31538 | 17.94294 | | 0.001 |
| PC (31:1) | C_39_H_77_O_8_N_1_P_1_ | 1.30721 | 0.49687 | | ＜0.001 |
| PE (16:0/18:1) | C_39_H_77_O_8_N_1_P_1_ | 1.30316 | 0.49834 | | 0.001 |
| PE (16:0p/18:1) | C_39_H_77_O_7_N_1_P_1_ | 1.28693 | 0.42976 | | ＜0.001 |
| SM (d22:1/18:0) | C_45_H_92_O_6_N_2_P_1_ | 1.27435 | 1.25240 | | 0.002 |
| PE (18:0/20:2) | C_43_H_83_O_8_N_1_P_1_ | 1.27374 | 0.54820 | | 0.002 |
| dMePE (16:0/18:2) | C_41_H_77_O_8_N_1_P_1_ | 1.27351 | 0.32562 | | ＜0.001 |
| PC (35:2) | C_43_H_83_O_8_N_1_P_1_ | 1.27335 | 0.54924 | | 0.001 |
| PE (18:0/18:2) | C_41_H_77_O_8_N_1_P_1_ | 1.27168 | 0.32610 | | ＜0.001 |
| PE (18:2/18:2) | C_41_H_75_O_8_N_1_P_1_ | 1.26425 | 0.20115 | | ＜0.001 |
| PC(33:4) | C_41_H_75_O_8_N_1_P_1_ | 1.25986 | 0.20668 | | ＜0.001 |
| PC(35:3) | C_43_H_81_O_8_N_1_P_1_ | 1.25633 | 0.32166 | | 0.001 |
| PE (16:0p/22:4) | C_43_H_79_O_7_N_1_P_1_ | 1.22876 | 0.42601 | | ＜0.001 |
| LPC(18:2) | C_26_H_51_O_7_N_1_P_1_ | 1.22339 | 3.52555 | | 0.007 |
| PC(36:0) | C_44_H_89_O_8_N_1_P_1_ | 1.20467 | 2.31755 | | ＜0.001 |
| LPC(20:3) | C_28_H_53_O_7_N_1_P_1_ | 1.19363 | 42.11043 | | ＜0.001 |
| PC(38:6) | C_46_H_81_O_8_N_1_P_1_ | 1.16031 | 0.61227 | | 0.002 |
| PC (18:1/18:2) | C_44_H_82_O_8_N_1_P_1_Na_1_ | 1.1579 | 0.61893 | | 0.002 |
| PC(38:5) | C_46_H_83_O_8_N_1_P_1_ | 1.14112 | 0.59665 | | 0.002 |
| PC (18:0e/18:2) | C_44_H_87_O_7_N_1_P_1_ | 1.13902 | 0.72873 | | 0.001 |
| DG (18:0/16:0) | C_37_H_76_O_5_N_1_ | 1.12291 | 1.48778 | | 0.002 |
| FA(20:4) | C_20_H_31_O_2_ | 1.10282 | 1.91189 | | 0.004 |
| PC (18:1/18:1) | C_44_H_84_O_8_N_1_P_1_Na_1_ | 1.08483 | 0.64047 | | 0.005 |
| PC (18:0p/18:2) | C_44_H_85_O_7_N_1_P_1_ | 1.06105 | 0.63437 | | 0.001 |
| SM (d18:0/18:1) | C_41_H_84_O_6_N_2_P_1_ | 1.05413 | 1.31144 | | 0.001 |
| PC(34:3) | C_42_H_79_O_8_N_1_P_1_ | 1.0425 | 1.91652 | | 0.003 |
| PE (18:0/18:1) | C_41_H_79_O_8_N_1_P_1_ | 1.02854 | 0.42583 | | 0.013 |

Table S7 Lipid species active reactions (dCP1 treatment vs control)

| Reaction chain | Z-score | Predicted genes |
| --- | --- | --- |
| PE(38:2) →PC(38:2) →DG(38:2) | 6.259 | *PEMT* |
| PS(36:2) →PE(36:2) →PC(36:2) →DG(36:2) | 6.219 | *PISD, PEMT* |
| PE(36:2) → PC(36:2) → DG(36:2) | 5.839 | *PEMT* |
| PE(36:3) → PC(36:3) → DG(36:3) | 5.703 | *PEMT* |
| PE(38:3) → PC(38:3) → DG(38:3) | 5.411 | *PEMT* |
| PC(38:3) → DG(38:3) | 4.35 | / |
| PC(36:3) → DG(36:3) | 4.104 | / |
| PE(36:4) → PC(36:4) | 4.089 | *PEMT* |
| PC(38:2) → DG(38:2) | 3.971 | / |
| PE(34:0) → PC(34:0) → DG(34:0) | 3.913 | *PEMT* |
| P-PE(36:2) → P-PC(36:2) | 3.871 | *CEPT1, PLD1, PLPP1, PLPP2, CHPT1* |
| PC(36:2) → DG(36:2) | 3.453 | / |

Table S8 Lipid species suppressed reactions (dCP1 treatment vs control)

| Reaction chain | Z-score | Predicted genes |
| --- | --- | --- |
| PC(36:2) → PS(36:2) | 4.476 | *PTDSS1* |
| DG(34:1) → PE(34:1) | 4.305 | *CEPT1* |
| DG(36:1)→PE(36:1)→PS(36:1) | 3.858 | *CEPT1, PTDSS2* |
| PE(36:2) → PS(36:2) | 3.777 | *PTDSS2* |
| DG(38:2) → PE(38:2) | 3.747 | *CEPT1* |
| DG(38:3) → PE(38:3) | 3.737 | *CEPT1* |
| DG(38:2) → PC(38:2) | 3.607 | *CHPT1* |
| DG(34:0) → PE(34:0) | 3.558 | *CEPT1* |
| DG(36:3) → PC(36:3) | 3.5 | *CHPT1* |
| DG(36:2)→PC(36:2)→PS(36:2)→PE(36:2) | 3.49 | *CHPT1, PTDSS1, PISD* |
| DG(36:2) → PE(36:2) | 3.416 | *CEPT1* |
| DG(38:3) → PC(38:3) | 3.41 | *CHPT1* |
| DG(36:3) → PE(36:3) | 3.06 | *CEPT1* |
| PE(36:1) → PS(36:1) | 1.949 | *PTDSS2* |

**Supplementary-Figures**


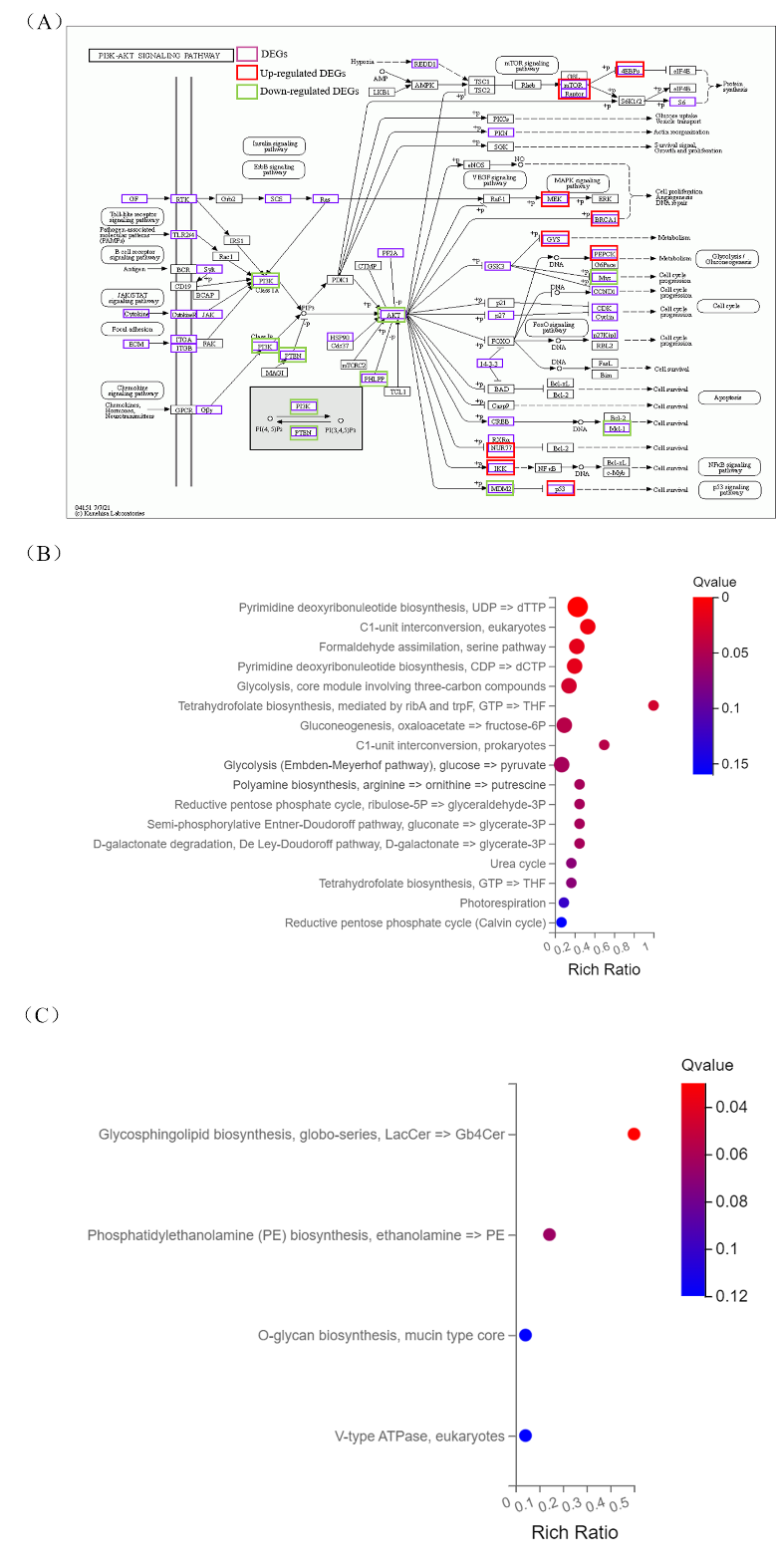


**Figure S1** KEGG Pathway and KEGG Module analysis. **(A)** PI3K-AKT pathway changes in M2-like TAM after treatment with the glycopeptide dCP1; **(B)** Up-regulated DEGs KEGG Module analysis; **(C)** Down-regulated DEGs KEGG Module analysis.


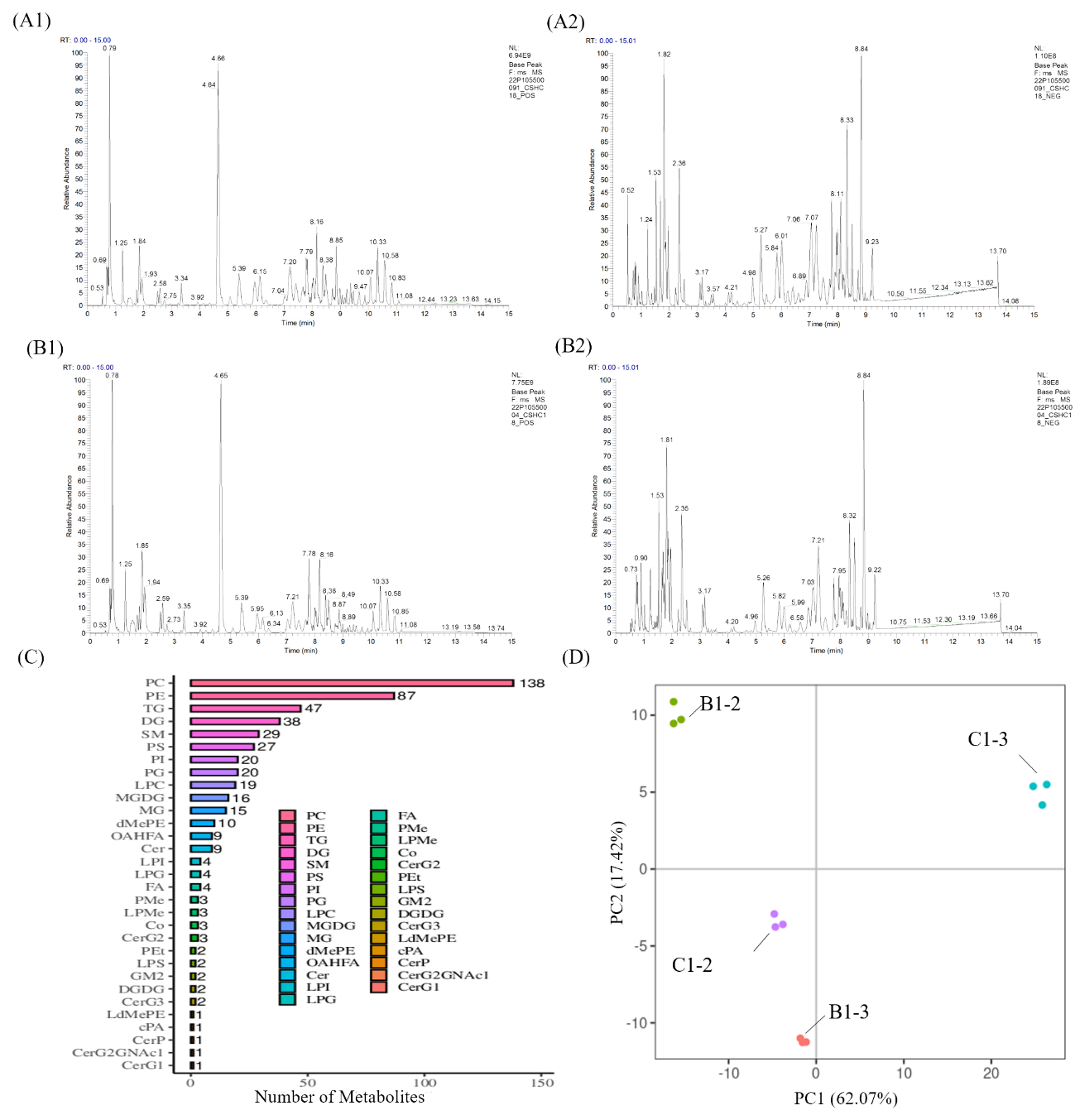


**Figure S2** Typical base peak chromatograms of experimental groups, principal component analysis, and classification of metabolites. **(A1)** B1-3 group positive ion mode; **(A2)** B1-3 group negative ion mode; **(B1)** C1-3 group positive ion mode; **(B2)** C1-3 group negative ion mode; **(C)** Metabolite classification; **(D)** PCA analysis.


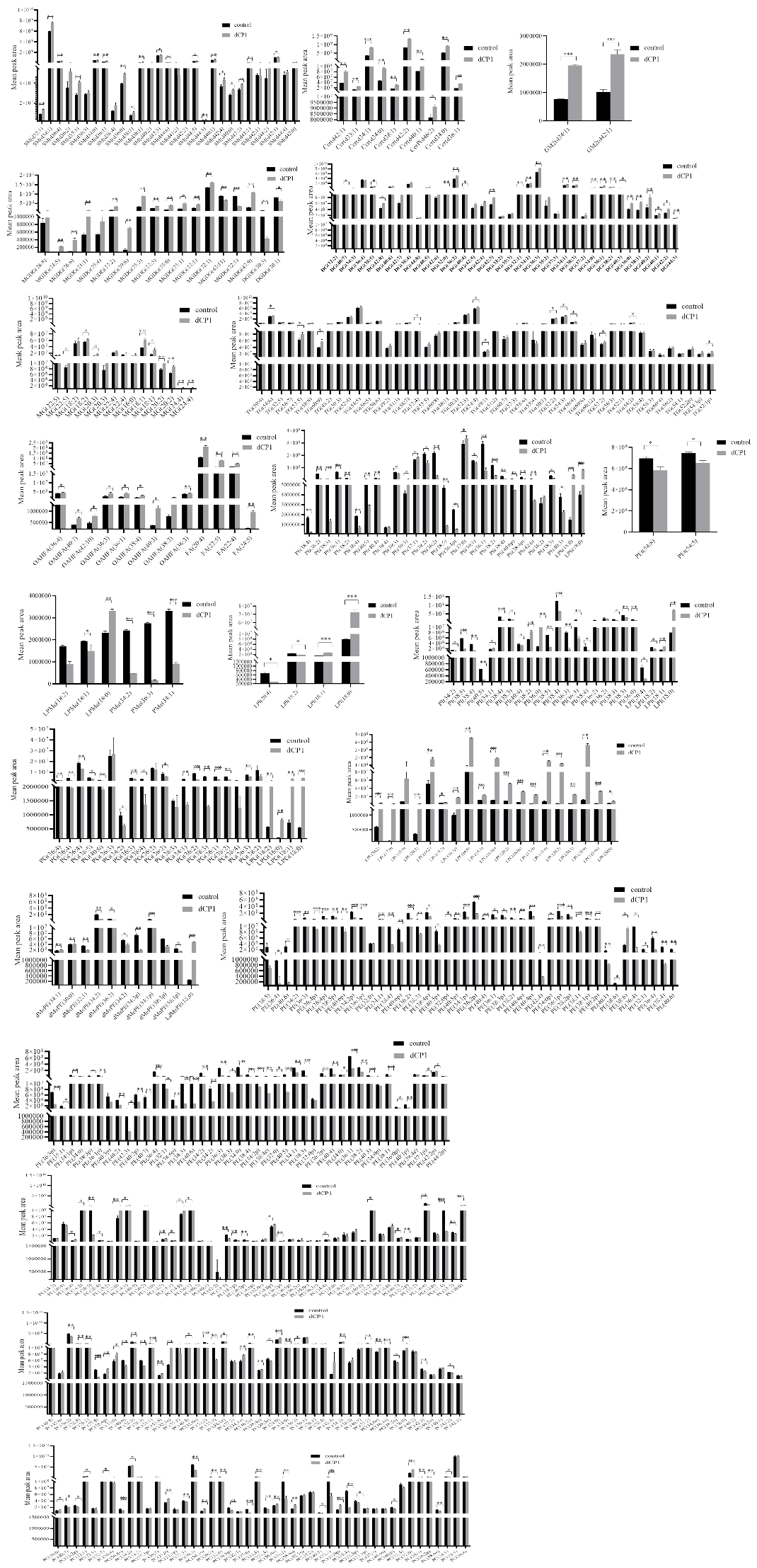


**Figure S3** Changes in lipid metabolism of M2-like TAM before and after treatment with dCP1.

* indicates *p* < 0.05, ** indicates *p* < 0.01, *** indicates *p* < 0.001


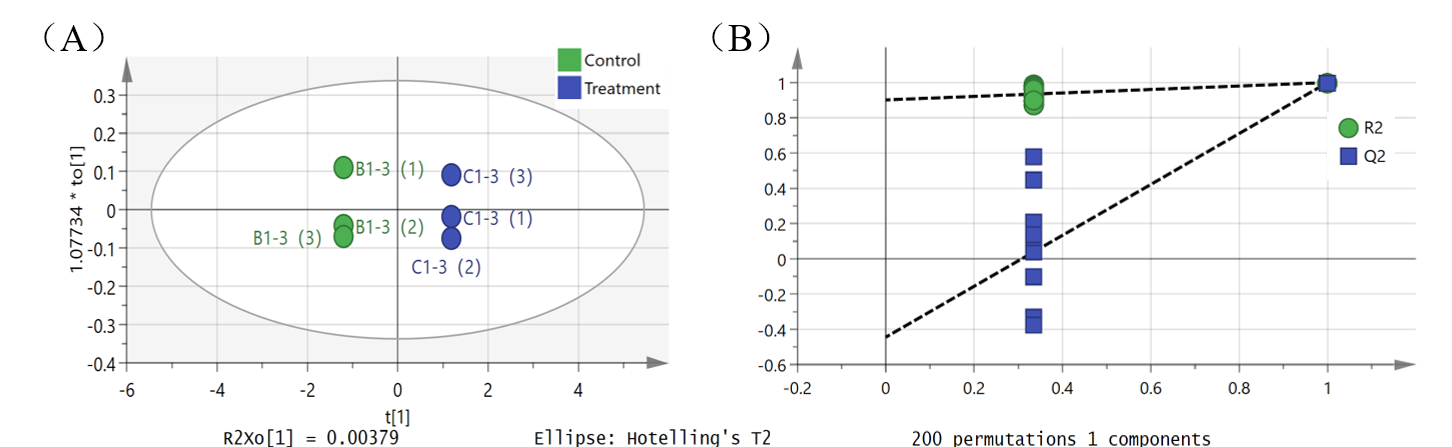


**Figure S4** OPLS-DA analysis of differential lipid metabolites

**(A)** OPLS-DA model score; **(B)** Permutation test of OPLS-DA model


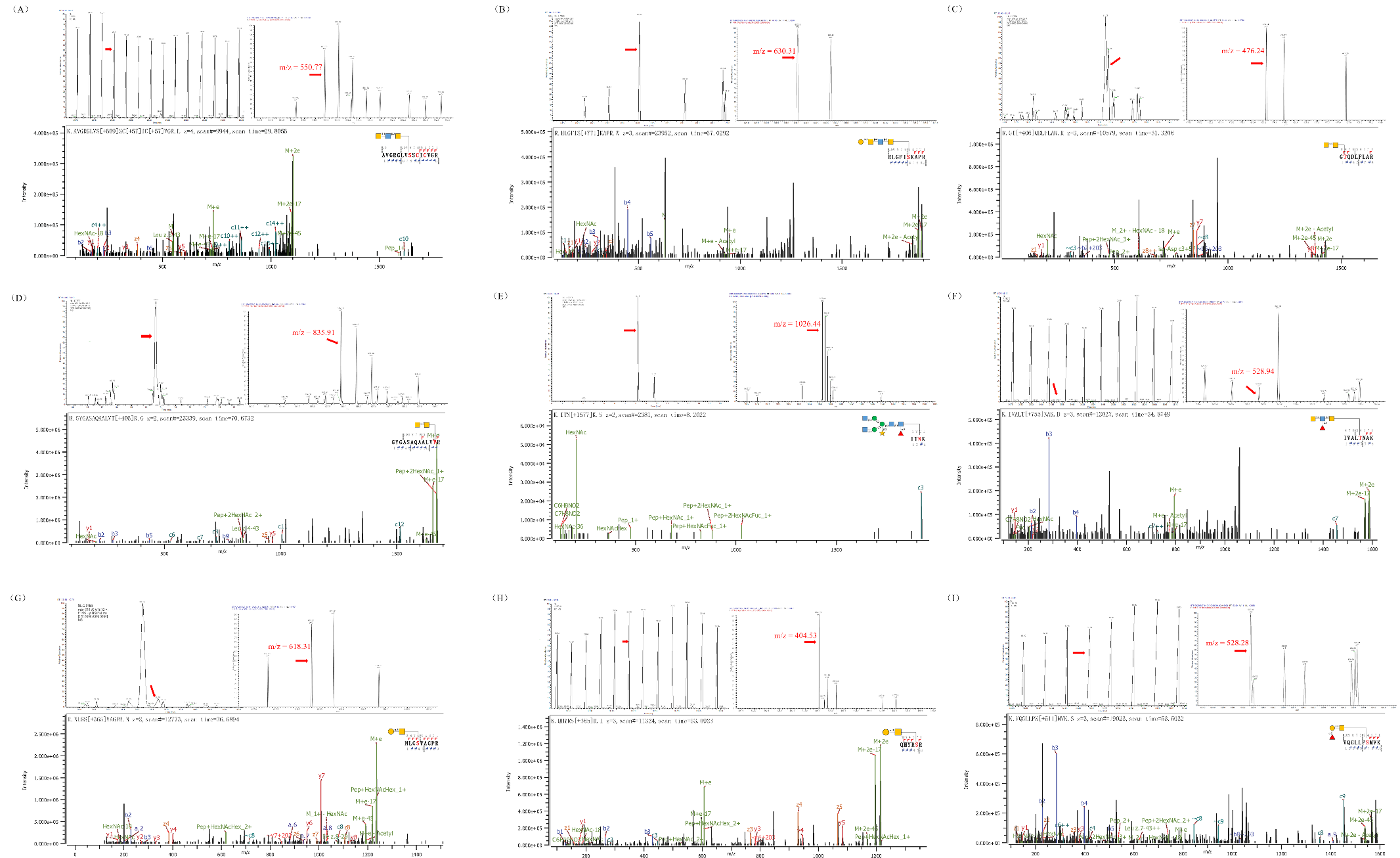


**Figure S5** The extracted ion chromatogram and mass spectra of the first 9 glycopeptides in dCP1 with high abundance and the highest confidence level. **(A)** The extracted-ion chromatogram, MS spectrum, and MS/MS spectrum of AVGRGLVS*SCICVGR; **(B)** The extracted-ion chromatogram, MS spectrum, and MS/MS spectrum of ELGFIS*KAPR; **(C)** The extracted-ion chromatogram, MS spectrum, and MS/MS spectrum of GT*QDLFLAR; **(D)** The extracted-ion chromatogram, MS spectrum, and MS/MS spectrum of GYGASAQAALVT*R; **(E)** The extracted-ion chromatogram, MS spectrum, and MS/MS spectrum of ITN*K; **(F)** The extracted-ion chromatogram, MS spectrum, and MS/MS spectrum of IVALT*NAK; **(G)** The extracted-ion chromatogram, MS spectrum, and MS/MS spectrum of NLGS*VAGPR; **(H)** The extracted-ion chromatogram, MS spectrum, and MS/MS spectrum of QHYRS*R; **(I)** The extracted-ion chromatogram, MS spectrum, and MS/MS spectrum of VQGLLPS*MVK.


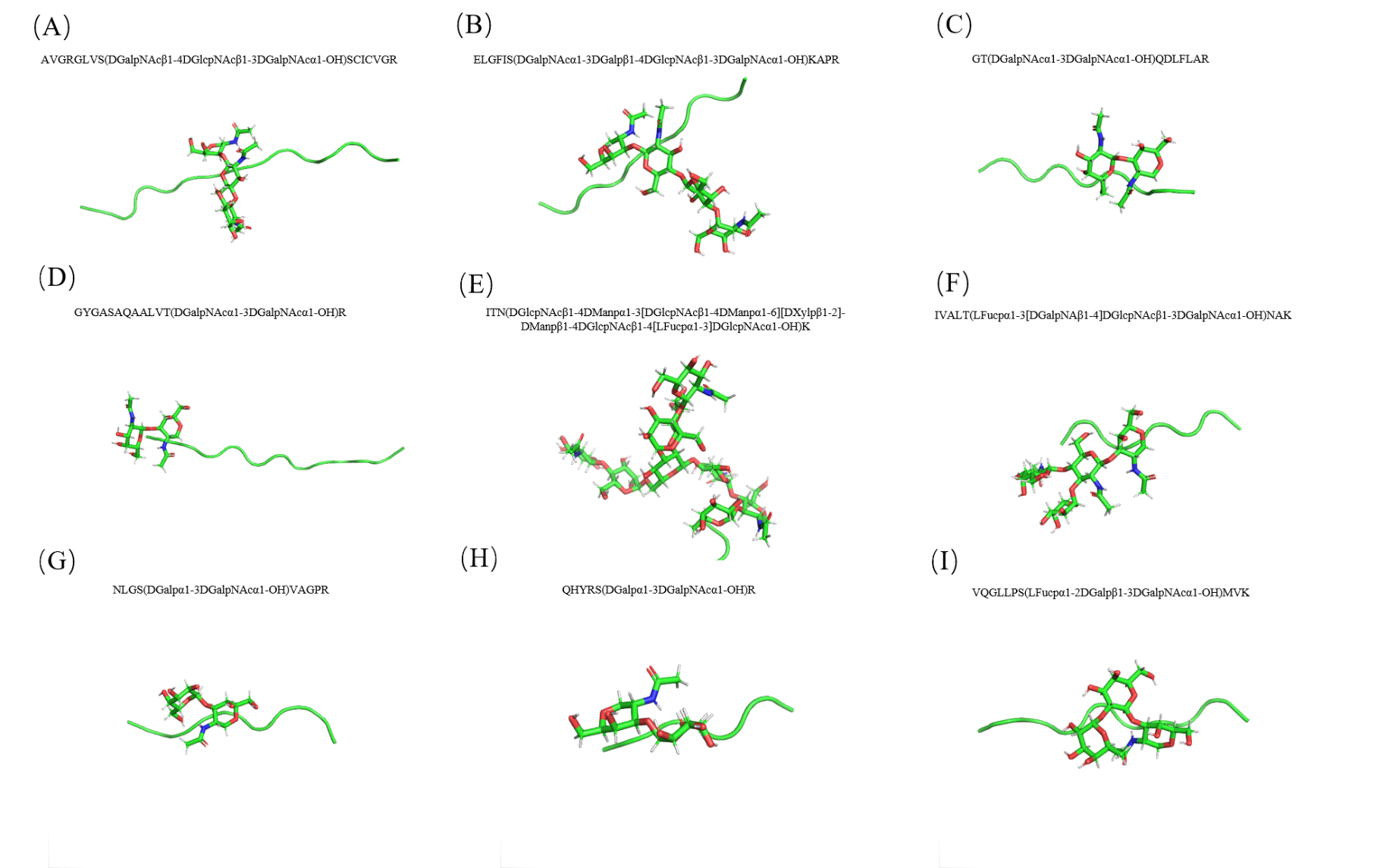


**Figure S6** Conformations of the glycopeptides


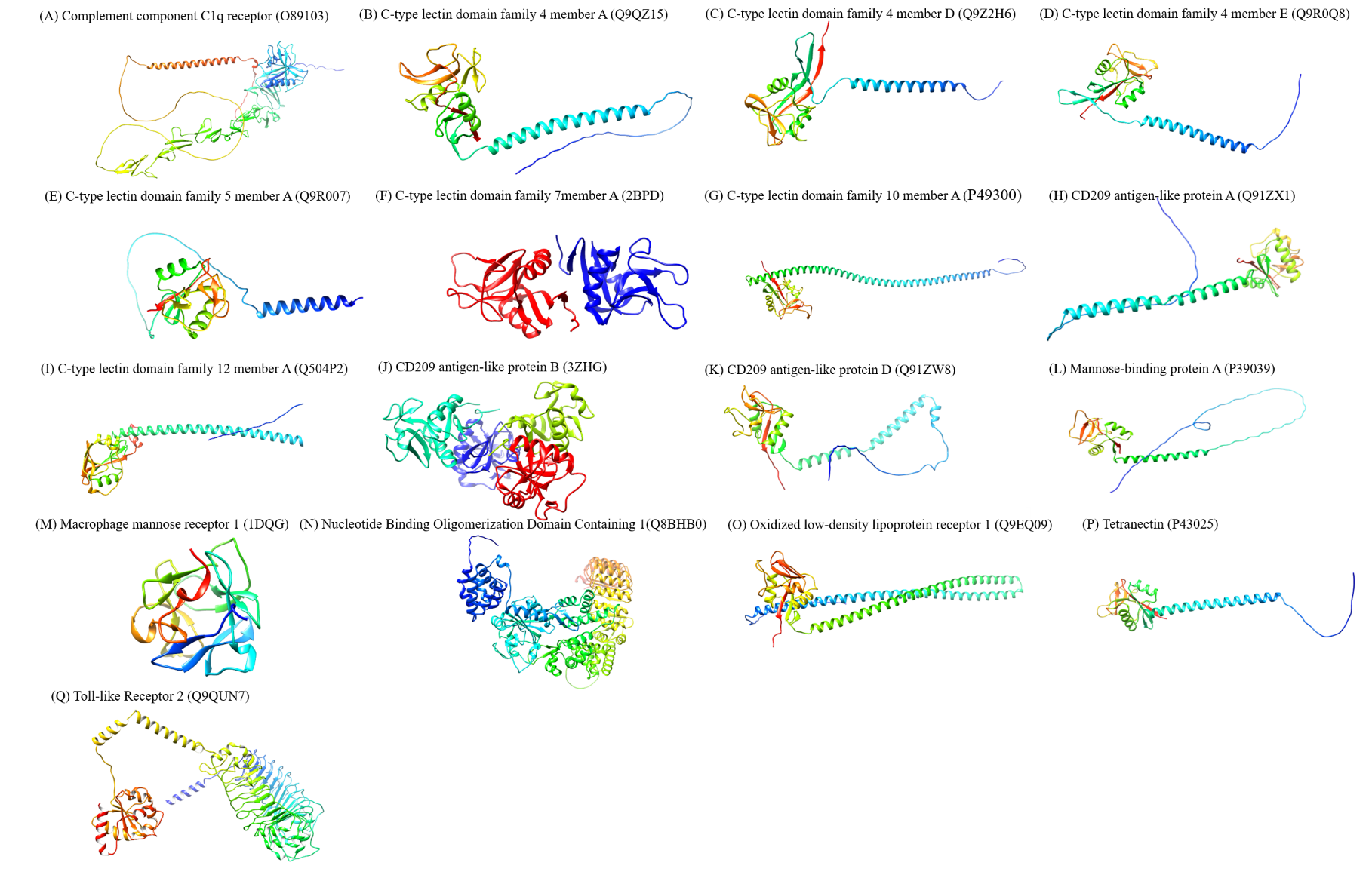


**Figure S7** Conformations of the receptors


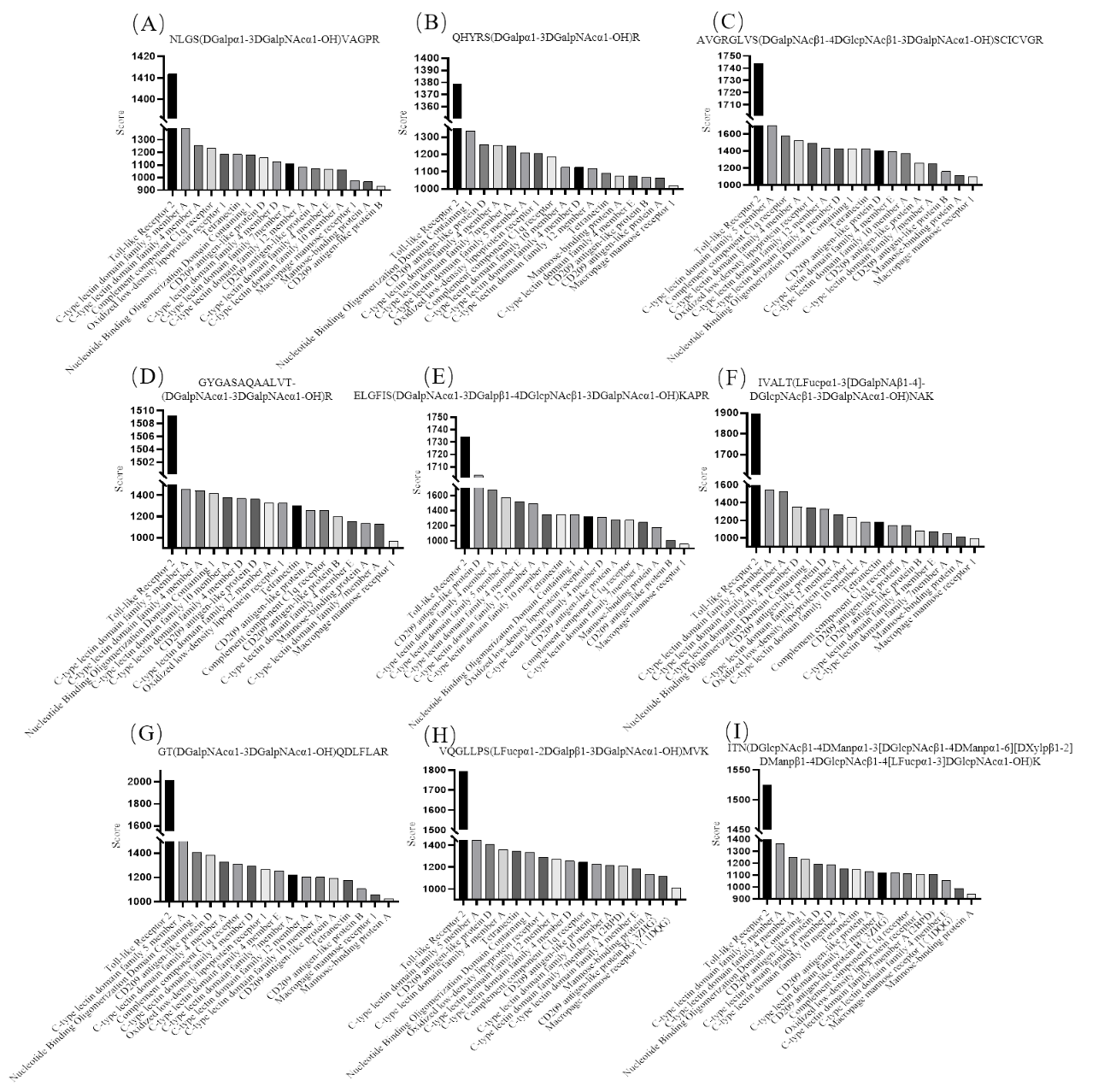


**Figure S8** Molecular docking score between glycopeptides and receptors
